# Enzyme-mediated depletion of l-cyst(e)ine synergizes with thioredoxin reductase inhibition for suppression of pancreatic tumor growth

**DOI:** 10.1038/s41698-019-0088-z

**Published:** 2019-06-03

**Authors:** Sabin Kshattry, Achinto Saha, Paul Gries, Stefano Tiziani, Everett Stone, George Georgiou, John DiGiovanni

**Affiliations:** 1 Division of Pharmacology and Toxicology, Dell Pediatric Research Institute, The University of Texas at Austin, 1400 Barbara Jordan Blvd, Austin, TX 78723 USA; 2 Department of Nutritional Sciences, Dell Pediatric Research Institute, The University of Texas at Austin, 1400 Barbara Jordan Blvd, Austin, TX 78723 USA; 3 Department of Molecular Biosciences, The University of Texas at Austin, Austin, Texas 78712 USA; 4 Department of Chemical Engineering, The University of Texas at Austin, Austin, Texas 78712 USA

**Keywords:** Pancreatic cancer, Biologics, Cancer therapy

## Abstract

Perturbing redox homeostasis potentially constitutes a selective cancer-killing strategy. An engineered human enzyme, cyst(e)inase that degrades extracellular cysteine (l-Cys) and cystine (CSSC) leading to depletion of intracellular l-Cys and glutathione (GSH) was evaluated for its effects on pancreatic cancer cell lines. Cyst(e)inase caused oxidative stress and apoptosis in only Panc1 cells, whereas MIA-PaCa2 and BxPC3 cells demonstrated survival under conditions of cyst(e)inase-mediated l-Cys depletion through maintenance of mitochondrial metabolism and lower levels of reactive oxygen species (ROS). A correlation was also observed between thioredoxin 1 protein levels and resistance to cyst(e)inase treatment. Notably, cyst(e)inase in combination with auranofin, a thioredoxin reductase inhibitor, caused a synergistic increase in mitochondrial ROS and apoptosis and inhibition of mitophagy in the more resistant cells. In addition, auranofin treatment sensitized the more resistant pancreatic cancer xenografts to cyst(e)inase without systemic toxicity. These data provide strong rationale to further investigate therapeutic strategies that target multiple antioxidant pathways for treatment of pancreatic ductal adenocarcinoma.

## Introduction

Pancreatic ductal adenocarcinoma (PDAC) has a dismal 5-year survival rate at 8%^1^ despite our increased understanding of the underlying cancer biology and improved ability to perform complex surgical procedures.^2^ One major reason for this is that over 80% of patients present at a stage when surgical resection is no longer possible. The only treatment option remaining is chemotherapeutic regimens, most of which provide only a modest survival increase and are associated with many toxicities.^3^ Notably, targeting KRAS, the driver oncogene in PDAC,^4^ has not been a clinically fruitful endeavor to date, and there is some evidence indicating that even its extinction might not be very therapeutically effective.^5,6^

A potentially powerful approach for selectively targeting cancer cells is by disrupting the intricate balance between reactive oxygen species (ROS) and antioxidants.^7–11^ This is especially relevant in the context of pancreatic cancer. Oncogenic Kras-mediated rewiring of mitochondrial metabolism in pancreatic cancer has been shown to be crucial in maintaining redox homeostasis.^12^ Surprisingly however, a subset of pancreatic cancer cells that are able to survive oncogenic Kras ablation actually have increased tumorigenic potential due to possession of stem-like properties and robust expression of genes involved in mitochondrial function and autophagy.^6^ Moreover, compared to other cancers, pancreatic cancer generally exhibits increased autophagy, which, by recycling dysfunctional mitochondria (mitophagy), helps curb ROS levels and maintain proper mitochondrial metabolism.^13,14^ The balance between ROS levels and mitochondrial functional status could provide a therapeutically exploitable target.

Recently, we reported^10^ that administration of an engineered human cyst(e)inase which depletes the extracellular pool of l-Cys and CSSC (collectively referred to as cyst(e)ine) leads to complete depletion of intracellular GSH, oxidative stress and selective cancer cell cytotoxicity in multiple murine tumor models. In the current work, we evaluated cyst(e)ine depletion as a potential therapeutic modality for pancreatic cancer treatment. Notably, we found that cyst(e)ine depletion was not cytotoxic to a subset of “resistant” pancreatic cancer cells. Further mechanistic studies revealed that resistant cells have high levels of thioredoxin 1 and also maintained mitochondrial metabolism and fitness. These findings suggested pathways for combinatorial approaches to overcome resistance. Concurrent administration of cyst(e)inase with auranofin, a thioredoxin reductase inhibitor and an FDA-approved drug resulted in sensitization of cyst(e)inase-resistant tumors without overt signs of systemic toxicity. Thus, targeting multiple antioxidant pathways shows promise for further study as a therapeutic modality for PDAC.

## Results

### Differential sensitivity of PDAC cell lines to l-Cys/CSSC deprivation

**Fig. 1:**
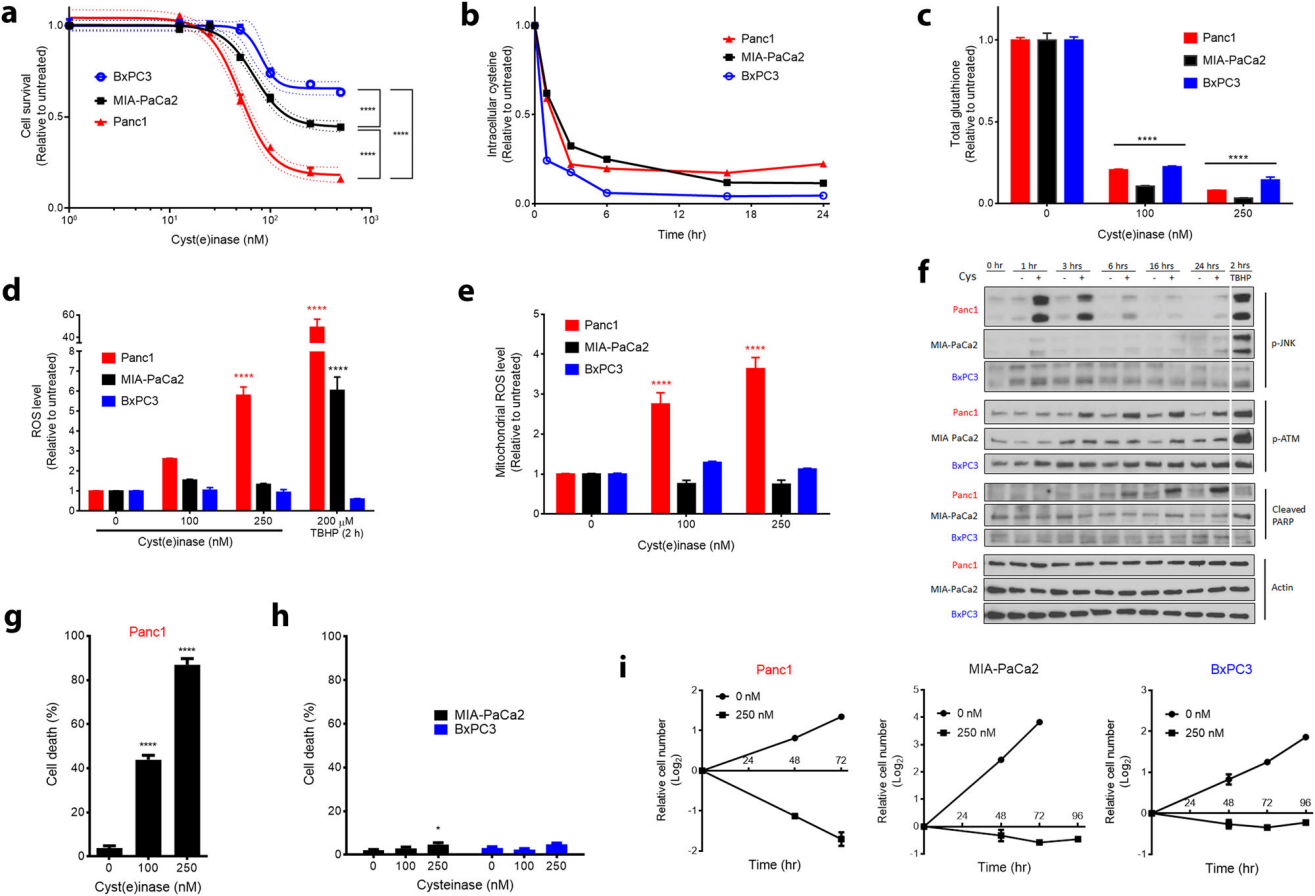
Effect of cyst(e)inase treatment on survival of PDAC cell lines. **a** Relative cell survival 48 h after cyst(e)inase treatment in three pancreatic cancer cell lines (*n* = 3 cultures for each dose). **b** Intracellular l-Cys levels upon treatment with 250 nM cyst(e)inase (one treated and one untreated culture for each time point, data from a representative experiment). **c** Intracellular GSH levels 24 h after treatment (*n* = 3 cultures for each dose). **d** Cellular ROS levels as assessed by 2′,7′-dichlorofluorescin diacetate (DCFDA) fluorescence 24 h after treatment. *tert*-Butyl hydrogen peroxide (TBHP) treatment is included as a positive control for ROS accumulation (*n* = 3–5 independent experiments). **e** Mitochondrial ROS levels as assessed by MitoSOX fluorescence following 24 h cyst(e)inase treatment (*n* = 3–6 independent experiments). **f** Oxidative stress signaling via JNK and ATM, and apoptosis signaling following cyst(e)inase treatment. Cys cyst(e)inase. A white line is added to separate the TBHP treatment group with the rest of the time course data. Original blots can be found in the Supplementary Figures. **g** Cell death in Panc1 cells as assessed by trypan blue staining 48 h after treatment (*n* = 3 independent experiments). **h** Cell death in MIA-PaCa2 and BxPC3 cells 72 h after cyst(e)inase treatment (*n* = 3–4 independent experiments). **i** Cellular growth assay of Panc1, MIA-PaCa2, and BxPC3 cells (*n* = 3 cultures for each time point) with cyst(e)inase treatment. For **f**, “+” represents 250 nM cyst(e)inase treatment. All data represent mean ± s.e.m. except for **b**. Dotted line in **a** represents the 95% confidence band for the fitted curve (four-parameter logistic model). *P < 0.05, ****P < 0.0001; compared to untreated controls (**c**–**e**, **g**, **h**), or between the bottom plateaus of the fitted curve (**a**); two-way ANOVA (**c**–**e**) or one-way ANOVA (**a**, **g**, **h**) with Bonferroni’s method for multiple-comparison test. Experiments in **f** were repeated twice with similar results. All other experiments were repeated three times or more

Treatment of three pancreatic cancer cell lines, BxPC3 (Kras^WT^), MIA-PaCa2 (Kras^G12C^), and Panc1 (Kras^G12D^), with cyst(e)inase caused a dose-dependent inhibition of cell survival to different extents (Panc1 >> MIA-PaCa2 > BxPC3) (Fig. 1a). A concentration of 250 nM cyst(e)inase in the culture medium, that depletes intracellular l-Cys to low residual levels, achieved a near-complete depletion of intracellular GSH within 24 h in all three cell lines (Fig. 1b, c). Despite a marked depletion of GSH in all three cell lines, cyst(e)inase induced an increase in total cellular ROS (Fig. 1d) and mitochondrial superoxide (mitochondrial ROS, mROS) levels (Fig. 1e) only in Panc1 cells. A known consequence of ROS accumulation in pancreatic cancer cells is activating phosphorylation of c-Jun N-terminal kinase (JNK), which causes cell cycle arrest at G_2_/M and subsequently apoptosis.^11^ Cyst(e)inase treatment caused activation of JNK within 1 h of treatment in Panc1 cells, but not in the other two more resistant cell lines (Fig. 1f). JNK was activated by *tert*-butyl hydrogen peroxide (TBHP) in Panc1 and also MIA-PaCa2 but not in BxPC3 cells, which were resistant to ROS accumulation even with TBHP treatment (Fig. 1d, f). Another signaling event indicating oxidative stress is phosphorylation of the ataxia-telangiectasia mutated (ATM) protein kinase even in the absence of DNA double-strand breaks (DSBs).^15^ Cyst(e)inase treatment caused activation of ATM within 3 h of treatment and induced apoptosis within 6 h, as evidenced by cleavage of poly (ADP-ribose) polymerase (PARP), again only in Panc1 cells (Fig. 1f). As a result, while Panc1 cells exhibited more than 80% cell death within 48 h, MIA-PaCa2 and BxPC3 cells were able to halt proliferation and maintain cell mass even after 96 h of cyst(e)inase treatment (Fig. 1g–i). The differential effects of cyst(e)inase treatment between Panc1 cells and the more resistant MIA-PaCa2 and BxPC3 cell lines were specifically due to inherent differences in sensitivity to l-Cys/CSSC deprivation (Supplementary Fig. 1a–e).

**Fig. 2:**
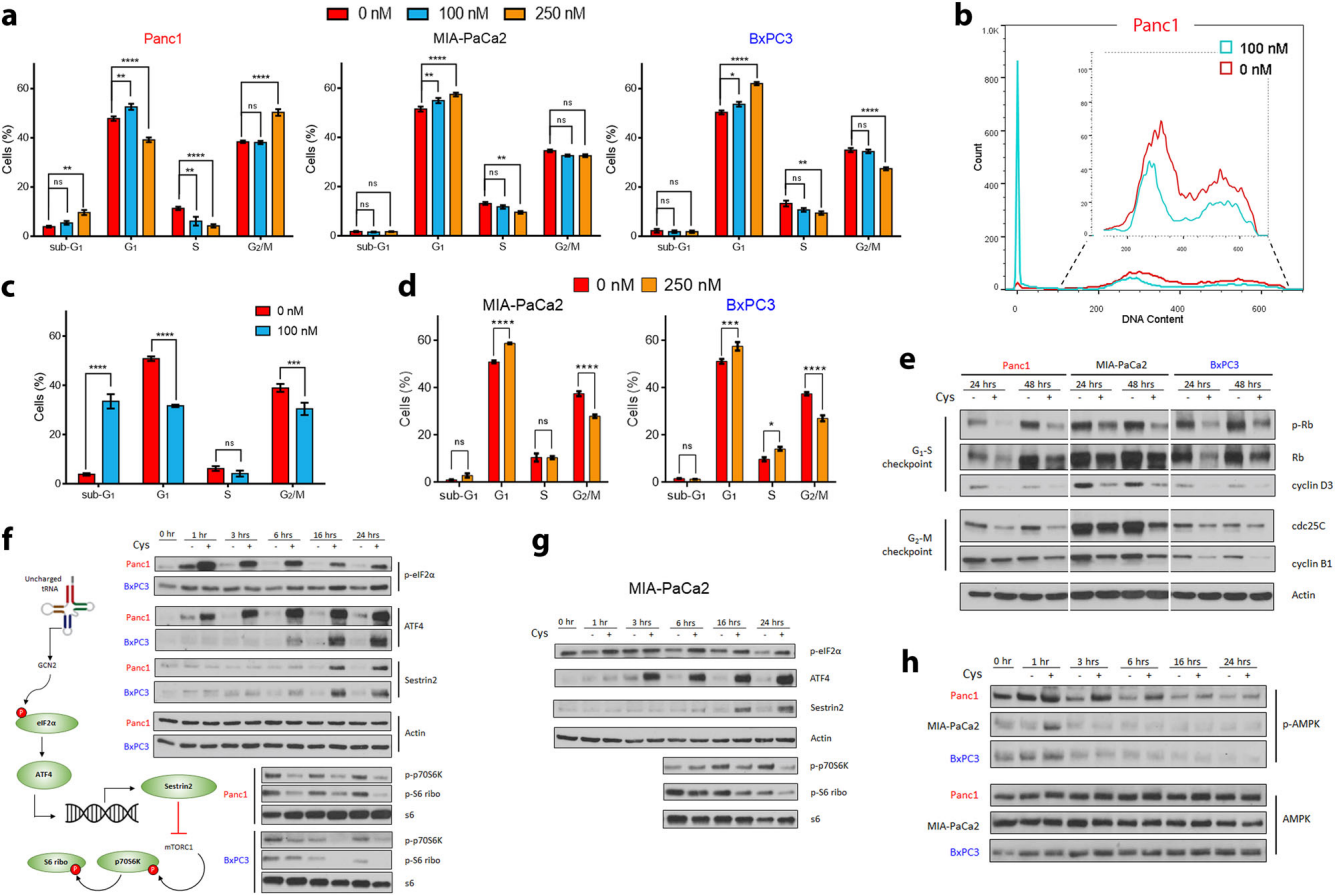
Effects of cyst(e)inase on cell cycle progression and stress signaling. **a** Quantification of cell cycle phase distribution 24 h after cyst(e)inase treatment (*n* = 3 independent experiments). **b** Cell cycle phase distribution 48 h after cyst(e)inase treatment for Panc1 cells. **c**, **d** Quantification of cell cycle phase distribution for Panc1 **c**, MIA-PaCa2 and BxPC3 cells **d** 48 h after treatment (*n* = 3 independent experiments). **e** Regulatory cell cycle proteins following 24 and 48 h cyst(e)inase treatment. **f**, **g** Effect of treatment on mTORC1 signaling via the eIF2α-ATF4-Sestrin2 pathway in Panc1, BxPC3 **f** and MIA-PaCa2 **g** cells. **h** Effect of treatment on AMPK signaling. For **e−h**, “+” represents 250 nM cyst(e)inase treatment except for **e**, where “+” for Panc1-48 h represents 100 nM treatment. All data represent mean ± s.e.m. *P < 0.05, **P < 0.01, ***P < 0.001, ****P < 0.0001, ns, not significant; compared to untreated controls; two-way ANOVA with Bonferroni’s method for multiple-comparison test. Experiment in (**e**−**h**) were repeated twice with similar results. All other experiments were repeated three times

Cyst(e)inase-treated Panc1 cells exhibited a G_1_ arrest as well as G_2_/M arrest and an apoptotic sub-G_1_ phase at higher cyst(e)inase concentrations (250 nM) after 24 h of treatment. In contrast, MIA-PaCa2 and BxPC3 which, as discussed above had lower ROS levels, underwent only G_1_ arrest (Fig. 2a). At 48 h following treatment with a lower dose of 100 nM cyst(e)inase, apoptotic sub-G_1_ phase Panc1 cells accounted for ~35% of the total cells, whereas MIA-PaCa2 and BxPC3 cells exhibited only G_1_ arrest with a subsequent failure to reach G_2_/M. (Fig. 2b–d). Cell cycle proteins regulating the checkpoint for G_1_-S transition and G_2_-M transition decreased for all cell lines with treatment (Fig. 2e).

Growth arrest following cyst(e)inase treatment could be a consequence of nutrient deprivation since l-Cys is required for protein synthesis. Mammalian cells have evolved two major distinct amino acid sensing mechanisms—the mechanistic target of rapamycin complex 1 (mTORC1) signaling pathway is activated during amino acid replete conditions to stimulate protein synthesis while the general control nonderepressible 2 (GCN2) pathway is activated during amino acid starvation to inhibit protein translation. Specifically, GCN2, which is activated by uncharged tRNAs, links the deprivation of any single amino acid to inhibition of mTORC1.^16^ Activated GCN2 phosphorylates eIF2α, which globally inhibits cap-dependent protein translation except for the translation of a few stress response transcription factors including ATF4, which transcriptionally upregulates Sestrin2. Sestrin2 inhibits the lysosomal localization of mTORC1, which is needed for its activation and subsequent phosphorylation of its downstream target p70S6K. Cyst(e)inase treatment activated the eIF2α-ATF4-Sestrin2 axis and inhibited the mTORC1 signaling pathway in all three cell lines (Fig. 2f, g). In the context of cyst(e)inase treatment, GCN2 signaling appeared more important than signaling through AMP kinase (AMPK), another known inhibitor of mTORC1, because sustained AMPK activation was observed in only Panc1 cells even though mTORC1 was inhibited in all three cell lines (Fig. 2h).

To further delineate whether the growth inhibitory effects of cyst(e)inase were due to GSH depletion and mROS production, we examined the effect of buthionine sulfoximine (BSO), a well-established inhibitor of GSH synthesis. Even though intracellular GSH was completely depleted by BSO within 24 h in all three cell lines, cell survival was unaffected (Supplementary Fig. 2a, b). BSO treatment induced an increase in cellular ROS levels (measured by DCFDA) in all three cells lines which was different than that observed after cyst(e)inase treatment (Supplementary Fig. 2c, compare with Fig. 1d). Importantly, BSO treatment did not increase mROS in Panc1 cells in contrast to the effect of cyst(e)inase treatment (Supplementary Fig. 2d, compare with Fig. 1e). These data clearly point to the important role of mROS in the effects of cyst(e)inase treatment on survival of the PDAC cells used in the current study.

To determine if the differences in cell survival upon cyst(e)inase treatment seen in cultured PDAC cell lines could be recapitulated in vivo, preclinical studies with cyst(e)inase were performed using all three pancreatic cancer cell lines in xenograft models. For these experiments, male nude mice harboring PDAC xenografts received biweekly intraperitoneal cyst(e)inase treatment (100 mg/kg). This dosing regimen of cyst(e)inase was previously shown to result in dramatic and sustained reduction of the blood levels of both l-cystine and l-cysteine and to inhibit growth of tumors in allograft, xenograft and spontaneous tumor models.^10^ As shown in Supplementary Fig. 3, the in vivo effect of cyst(e)inase on reducing xenograft tumor growth mirrored the in vitro sensitivity profile of the three pancreatic cell lines. Panc1 exhibited the most growth inhibition, the growth of MIA-PaCa2 tumors was inhibited to a lower extent whereas BxPC3 xenografts were completely resistant.

### Impact of cyst(e)inase treatment on mitochondrial fitness

**Fig. 3:**
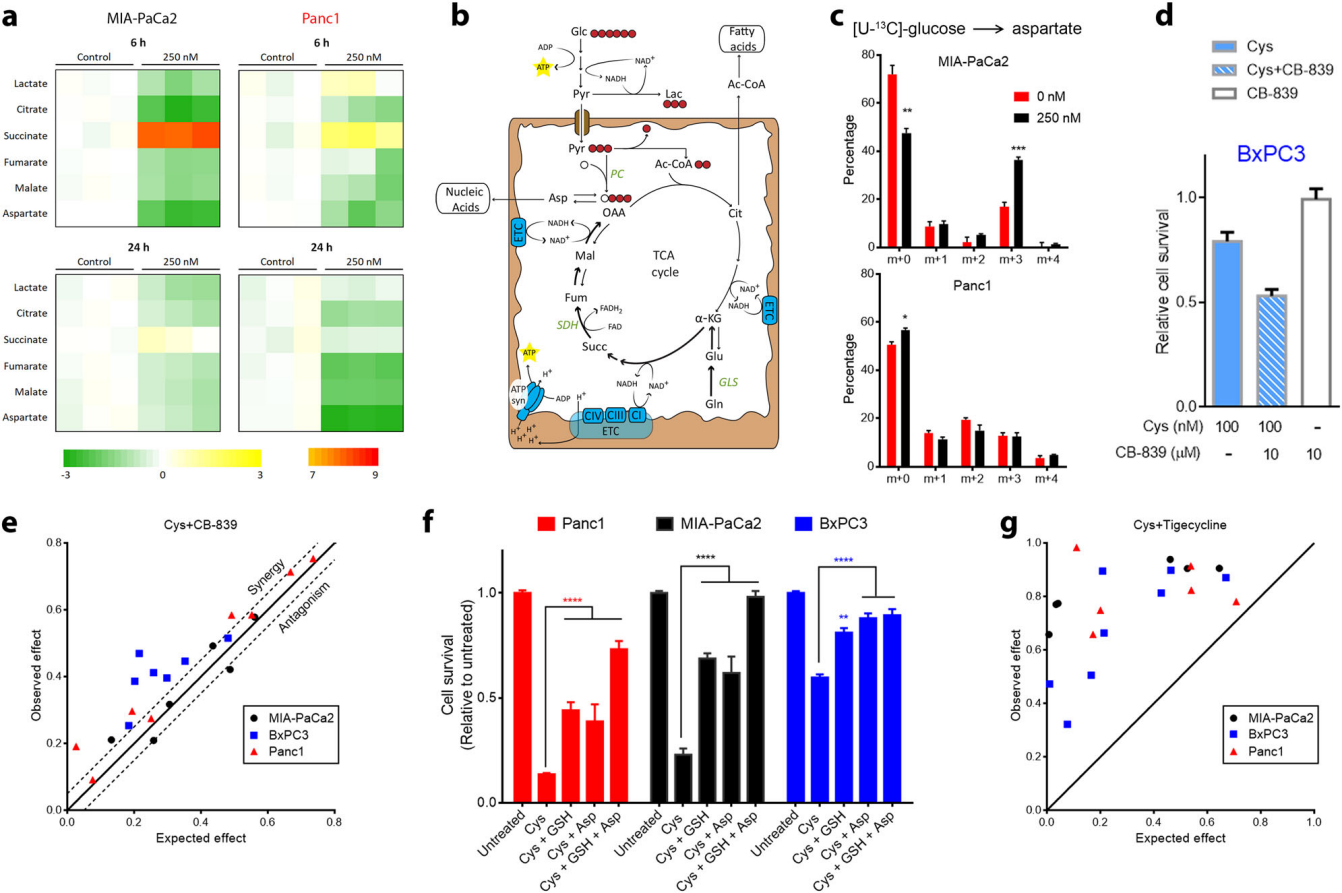
Resistant cells maintain anaplerotic capacity during cyst(e)inase treatment making mitochondrial metabolism a synergistic target. **a** TCA cycle intermediates and related metabolite levels 6 and 24 h after 250 nM cyst(e)inase treatment in MIA-PaCa2 and Panc1 cells (*n* = 3 cultures). Color bar shows Log_2_ scale. **b** Predicted induction of pyruvate carboxylase (PC) activity by MIA-PaCa2 cells to replenish pools of oxaloacetate (OAA) and subsequently aspartate (Asp) via [U-^13^C]-glucose. Glc glucose, Pyr pyruvate, Lac lactate, Ac-CoA acetyl-CoA, Cit citrate, α-KG α-ketoglutarate, Glu glutamate, Gln glutamine, Succ succinate Fum fumarate, Mal malate, GLS glutaminase, SDH succinate dehydrogenase, ETC electron transport chain, ATP syn ATP synthase. **c** Mass isotopologue analysis of aspartate in MIA-PaCa2 (top) and Panc1 (bottom) cells cultured in [U-^13^C]-glucose and treated with cyst(e)inase for 6 h (*n* = 3 cultures). **d** Cell survival of BxPC3 cells treated with combination of cyst(e)inase (Cys) and CB-839 for 48 h. Values are relative to untreated control, which is not shown (*n* = 3 cultures for each condition). **e** Isobologram of the effect of the combination of cyst(e)inase and CB-839 (data from 3–4 independent experiments). Dashed line represents 5% error to distinguish true synergy from experimental variability. **f** Relative cell survival 48 h after treatment with either 200 nM Cys alone or in combination (single or double) with 0.5 mM GSH ethyl ester (GSH) and 5 mM methyl aspartate (Asp) (*n* = 3 independent experiments). **g** Isobologram of the effect of the combination of cyst(e)inase and tigecycline (data from 3–4 independent experiments). All data represent mean ± s.e.m. *P < 0.05, **P < 0.01, ***P < 0.001, ****P < 0.0001; compared to untreated controls **c** or to Cys treatment **f**; two-sided Student’s *t* test **c** or two-way ANOVA with Bonferroni’s method for multiple-comparison test **f**. All experiments were repeated (in exact or similar form) three times or more

As shown in Fig. 1, l-Cys/CSSC depletion caused mROS-mediated cytotoxicity in Panc1 cells, and induction of a nonproliferative phenotype in the other two resistant cell lines, which necessitates specific adaptions in nutrient utilization by the mitochondria.^17^ It has recently been shown that cancer cells can decrease their reliance on glutamine-mediated anaplerosis under conditions of decreased cystine availability.^18^ However, cells still need to maintain a mechanism for replenishing aspartate, most of which is derived from glutamine in cell culture,^19^ as it is a limiting metabolite for cell survival and proliferation.^20,21^ This is exacerbated by the fact that aspartate has poor cell permeability and intracellular synthesis is the only substantial biological source.^20,22^ Since the more resistant PDAC cells (MIA-PaCa2 and BxPC3) are able to maintain cell mass upon cystine depletion, differences in aspartate levels and anaplerotic capacity between the three PDAC cell lines were explored. GC-MS-based metabolomics analysis revealed that within 3 h after the addition of cyst(e)inase there was a marked increase in succinate levels in MIA-PaCa2 cells, with succinate returning to near pretreatment levels by 24 h. A more modest change in succinate was observed in Panc1 cells with no change seen in BxPC3 cells (Fig. 3a, Supplementary Fig. 4a, b). This finding suggested that cyst(e)inase treatment of MIA-PaCa2 cells likely produced a blockade of the tricarboxylic acid (TCA) cycle at succinate dehydrogenase (SDH, Complex II), which is downstream of glutamine-mediated anaplerosis and catalyzes the oxidative conversion of succinate to fumarate using the electron acceptor FAD. Blockade of the TCA cycle at SDH without a mechanism to replenish pools of downstream metabolites (i.e., oxaloacetate) significantly impairs aspartate synthesis and hence cell survival (Fig. 3b). However, cells that are deficient in SDH, or cells that overcome glutamine dependence require upregulation of pyruvate carboxylase (PC) to replenish pools of aspartate^23–25^ (Fig. 3b). The initial drop in aspartate levels following cyst(e)inase treatment eventually recovered in MIA-PaCa2 cells (to more than 50% of control) whereas it continued to drop in Panc1 cells (to less than 15% of control) (Fig. 3a). MIA-PaCa2 cells were able to recover their aspartate levels via anaplerosis through PC as evidenced by an increase in [U-^13^C]-glucose derived m + 3 fraction in aspartate and all the way to fumarate (Fig. 3c and Supplementary Fig. 4d). Following cyst(e)inase treatment, Panc1 cells became deficient in anaplerosis from both glucose and glutamine as seen from the absence of PC upregulation and decrease in proline, a glutamine-derived amino acid (Fig. 3c and Supplementary Fig. 4c).

In contrast, BxPC3 cells, which are completely resistant to cyst(e)inase treatment in vivo, were able to maintain aspartate levels at more than 50% of control upon cyst(e)inase treatment but without requiring upregulation of PC activity (Supplementary Fig. 4b, e). BxPC3 cells, at least partially, depended on anaplerosis through glutamine as combining cyst(e)inase with CB-839, a glutaminase (GLS) inhibitor, produced a mild combinatorial inhibition of cell survival (Fig. 3d). In fact, BxPC3 cells were the most sensitive out of the three lines to concurrent glutaminase inhibition (Fig. 3e). BxPC3 cells also appeared to have an increased capacity to rewire glutamine metabolism as evidenced by the fact that only these cells easily acquired anchorage independence and formed spheroids, a process that requires reductive carboxylation of glutamine-derived α-ketoglutarate for maintenance of mitochondrial redox homeostasis^26^ (Supplementary Fig. 4f). Inhibiting mitochondrial pyruvate transport with UK5099 had a mild combinatorial effect in all three cell lines (Supplementary Fig. 4g). Based on these data, anaplerotic sources other than glutamine and glucose that fuel aspartate synthesis in the resistant BxPC3 cells may also be involved and remain to be determined. Collectively, the data in Fig. 3a–e and Supplementary Fig. 4a–g demonstrate that both MIA-PaCa2 and BxPC3 cells are better able to maintain aspartate levels (albeit by different mechanisms) and cell survival during l-Cys/CSSC and GSH depletion compared to Panc1 cells.

In further experiments, supplementing cells treated with cyst(e)inase with cell-permeable forms of GSH and aspartate as single agents partially rescued cell survival in all three cell lines but the two in combination markedly augmented cell survival to untreated levels in MIA-PaCa2 and BxPC3 and near-untreated levels in Panc1 (Fig. 3f). The mechanism of rescue with GSH ethyl ester in MIA-PaCa2 and BxPC3, in which cyst(e)inase treatment did not induce oxidative stress remains to be determined. Possible mechanisms could be through normalization of redox sensitive protein signaling^27^ or cleavage of this GSH at the cell surface by gamma-glutamyl transpeptidase followed by passive diffusion of Cys-Gly-ethyl ester dipeptide inside the cell and subsequent hydrolysis to produce intracellular l-Cys.^28,29^ These data also provide further support for the importance of aspartate in cell survival in all three cell lines following treatment with cyst(e)inase.

A major requirement for the biosynthesis of aspartate is the proper functioning of the electron transport chain and maintenance of the NAD+/NADH ratio.^20,21^ The marked depletion of aspartate seen with cyst(e)inase treatment only in Panc1 cells was not due to a perturbation of this ratio since all three cell lines exhibited similar changes in this ratio with cyst(e)inase treatment, and combining cyst(e)inase with rotenone (inhibitor of Complex I, which recycles NADH into NAD+) induced only a mild combinatorial effect (Supplementary Fig. 4h, i). In addition, MIA-PaCa2 and BxPC3 cells were more sensitive to rotenone treatment suggesting that they have an increased basal reliance on the electron transport chain (Supplementary Fig. 4j). Further supporting the idea that Panc1 cells cannot maintain mitochondrial metabolism during l-Cys/CSSC deprivation was the observation that cyst(e)inase induced an increase in the glycolytic enzyme hexokinase with concomitant decrease in the mitochondrial enzymes pyruvate dehydrogenase (PDH) and succinate dehydrogenase-A (SDH-A) (Supplementary Fig. 4k). Interestingly, both of these mitochondrial flavoproteins, PDH and SDH-A, which are integral to mitochondrial energy production,^30,31^ are also known to be sites of ROS production.^32^ There was no significant difference between the three cell lines in basal reliance on ATP synthase as probed by its inhibitor oligomycin, but interestingly, MIA-PaCa2 and BxPC3 could be sensitized to cyst(e)inase through concurrent oligomycin treatment (Supplementary Fig. 4l) corroborating the notion that a quiescent, nonproliferative phenotype requires oxidative phosphorylation.^17^ Collectively, these data suggested that perturbing the biosynthetic and bioenergetic functions of the mitochondria could be a viable approach to obtain synergy with cyst(e)inase, which is most compellingly demonstrated by the fact that tigecycline, an approved antibiotic that inhibits mitochondrial protein translation, produced a strong synergistic effect with cyst(e)inase in all three cell lines in vitro (Fig. 3g). These data further support the hypothesis that resistance to cyst(e)inase treatment in certain PDAC cells is due, at least in part, to maintaining mitochondrial fitness.

### Inhibition of thioredoxin reductase synergizes with cyst(e)inase via mROS accumulation and mitophagy blockade

**Fig. 4:**
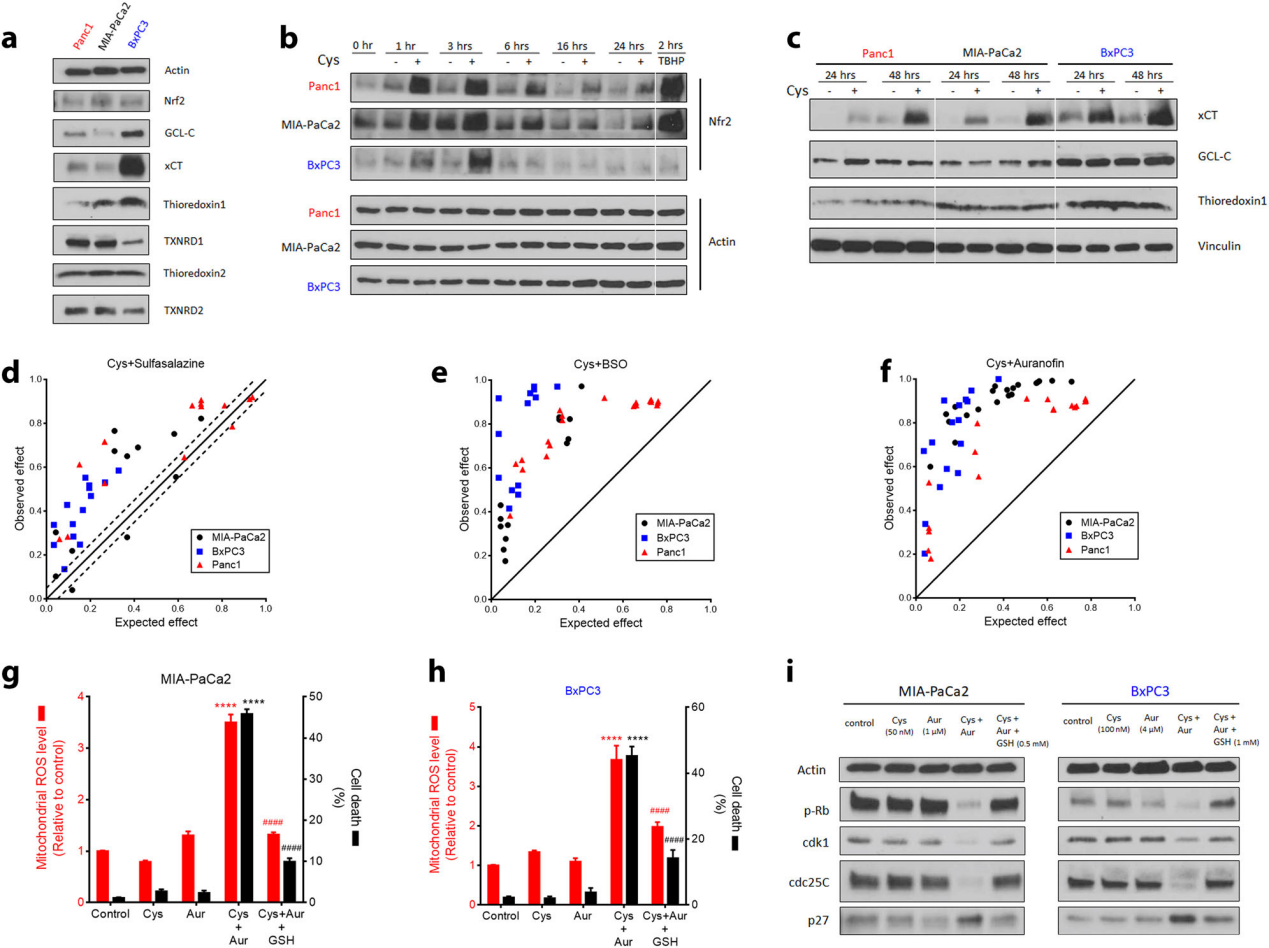
Expression of antioxidant proteins upon cyst(e)inase treatment and their concurrent inhibition. **a** Comparison of proteins related to antioxidant function in the three PDAC cell lines. Nrf2 nuclear factor (erythroid-derived 2)-like 2, GCL-C glutamate-cysteine ligase catalytic subunit, xCT cystine/glutamate antiporter, TXNRD1 and TXNRD2, thioredoxin reductase 1 and 2, respectively. **b**, **c** Expression of antioxidant proteins following cyst(e)inase treatment. TBHP treatment (200 μM, 2 h) is included as a positive control for ROS accumulation **b**. **d−f** Isobologram of the effect of the combination of cyst(e)inase (Cys) and sulfasalazine **d**, cyst(e)inase and BSO **e**, and cyst(e)inase and auranofin **f** (data from more than three independent experiments). **g** Relative mitochondrial ROS (mROS) levels (red) and cell death (black) in MIA-PaCa2 cells 24 h after treatment with 50 nM Cys, 0.5 μM (mROS) or 2 μM (cell death) auranofin (Aur), their combination (Cys + Aur), and the combination plus 0.5 mM GSH ethyl ester (GSH) (for mROS, *n* = 5 independent experiments; for cell death, *n* = 3 independent experiments). **h** Relative mitochondrial ROS (mROS) levels (red) and cell death (black) in BxPC3 cells 24 h after treatment with 100 nM Cys, 2 μM (mROS) or 4 μM (cell death) Aur, their combination (Cys + Aur), and the combination plus 0.5 mM GSH ethyl ester (GSH) (for mROS, *n* = 5 independent experiments; for cell death, *n* = 3 independent experiments). **i** Regulatory cell cycle proteins 24 h after the indicated combinatorial treatments in MIA-PaCa2 and BxPC3 cells. For (**b**, **c**), “+” represents 250 nM cyst(e)inase treatment except for (**c**), where “+” for Panc1–48 h represents 100 nM treatment. ####*P* or ****P < 0.0001; for (**g**, **h**), compared to untreated controls (*, comparison done for Cys, Aur and Cys + Aur) or to Cys + Aur (#, comparison done for only Cys + Aur + GSH); one-way ANOVA with Bonferroni’s method for multiple-comparison test. Experiments in (**a**, **b**, **c**, **i**) were performed twice. All other experiments were repeated three times or more

Based on the observations that MIA-PaCa2 and BxPC3 cells, unlike Panc1 cells, maintain mitochondrial fitness and have low levels of mitochondrial ROS during cyst(e)inase treatment, we further evaluated the possibility that concurrently inhibiting antioxidant pathways might synergize with l-Cys/CSSC and GSH depletion. Maintaining mitochondrial function in the face of cyst(e)inase-mediated GSH depletion requires other mechanisms for detoxification of ROS, the majority of which are produced by the ETC.^7^ Therefore, potential differences in antioxidant proteins between the three PDAC cell lines was examined. Basal levels of glutamate-cysteine ligase (GCL-C, the first enzyme involved in glutathione synthesis) were elevated in BxPC3 compared to the other cell lines but there was no correlation with sensitivity as MIA-Paca2 cells had the lowest protein level among the three cell lines. Cystine/glutamate antiporter (xCT), which imports cystine into the cell was also highly upregulated in BxPC3 but not in the other two cell lines (Fig. 4a). Basal levels of the transcription factor nuclear factor (erythroid-derived 2)-like 2 (Nrf2), a master regulator of cellular antioxidant response, were similar in the three cell lines (Fig. 4a). Notably, the level of thioredoxin 1 correlated with the sensitivity of the three PDAC cell lines to cyst(e)inase treatment.

The impact of cyst(e)inase treatment on levels of Nrf2, xCT, GCL-C and thioredoxin 1 was examined in all three lines. Nrf2 levels increased in all three cell lines but this response was more sustained in Panc1 and MIA-PaCa2 compared to BxPC3 (Fig. 4b); hence, Nrf2 levels did not correlate with sensitivity to cyst(e)inase treatment. Cystine deprivation is known to induce xCT in these particular cell lines,^33^ which was recapitulated by cyst(e)inase treatment and as a result, the difference in basal xCT expression between the cell lines was diminished following treatment with cyst(e)inase (Fig. 4c, compare with Fig. 4a). Cyst(e)inase treatment did not lead to induction of GCL-C or thioredoxin 1 although the differences in thioredoxin 1 protein levels were maintained in all three cell lines during cyst(e)inase treatment (BxPC3 > MIA-PaCa2 > Panc1) (Fig. 4c). Based on these results, drugs targeting several of these pathways were evaluated for combinatorial effects on cell survival together with cyst(e)inase. Concurrent inhibition of xCT using sulfasalazine and concurrent inhibition of GCL-C using BSO together with cyst(e)inase produced synergistic inhibition of cell survival in all three cell lines (Fig. 4d, e). The similar levels of synergy obtained in all three cell lines with cyst(e)inase plus sulfasalazine combination (even though basal xCT expression was highest in BxPC3) are likely due to the induction of xCT with cyst(e)ine depletion such that xCT was highly induced in all three cell lines by 48 h. The synergy observed with BSO is interesting and may reflect a greater depletion of intracellular GSH stores when combined with cyst(e)inase.

**Fig. 5:**
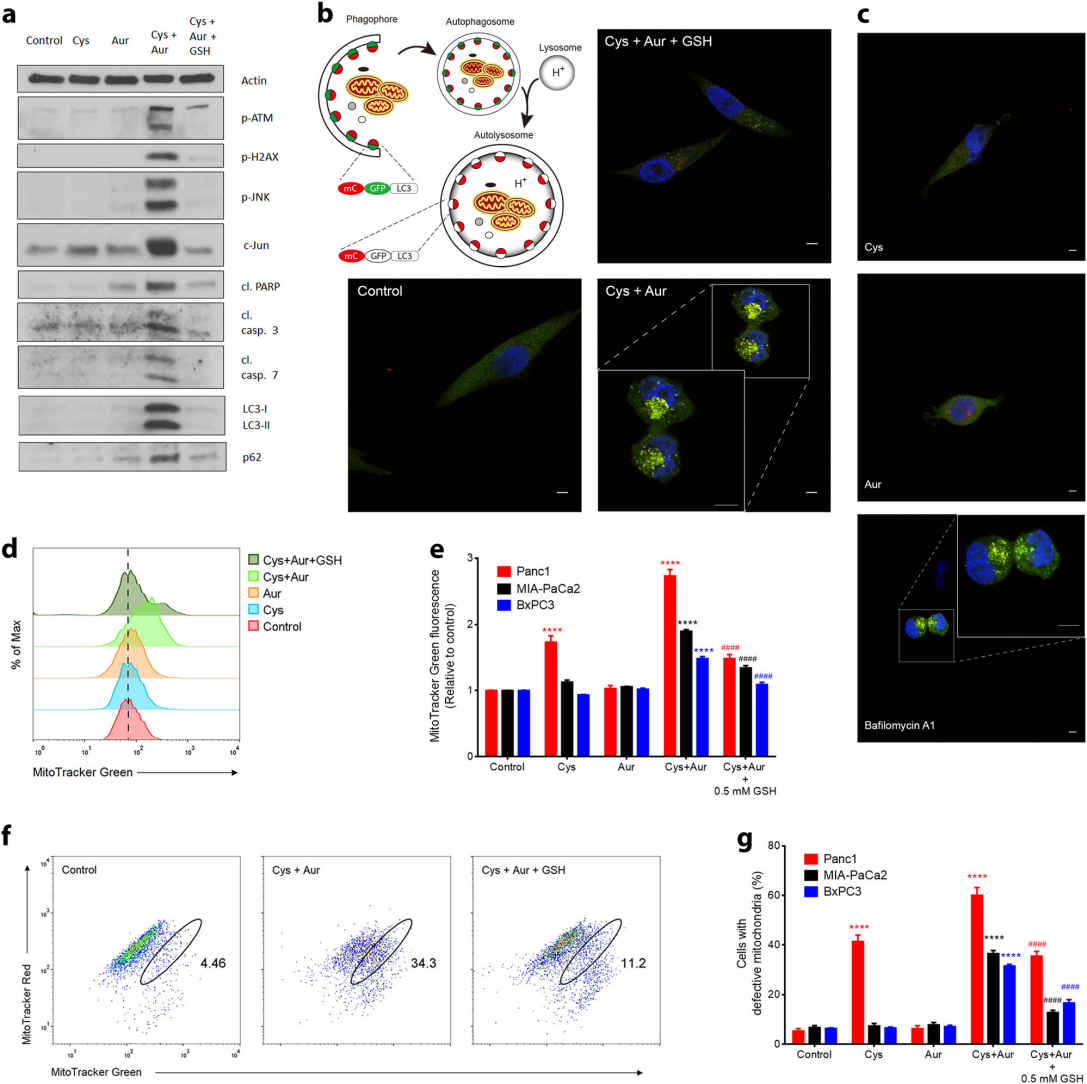
Effect of combining cyst(e)inase and auranofin on oxidative stress, DNA damage markers and mitophagy. **a** Oxidative stress markers, and apoptosis and autophagy signaling in MIA-PaCa2 cells treated as follows: 50 nM Cys, Aur: 1 or 2 μM, Cys + Aur combination, combination + 0.5 or 1 mM GSH ethyl ester (GSH). Only the cleaved (cl.) forms of PARP, caspase (casp.) 3 and caspase 7 are shown. **b** The doubly tagged mCherry-GFP-LC3 protein emits both red (mCherry) and green (GFP) fluorescence (yellow when merged) from autophagosomes but only red fluorescence after fusion with lysosome due to quenching of GFP in the acidic environment of the autolysosome (top left). Confocal microscopy images of mCherry-GFP-LC3-transfected MIA-PaCa2 cells 6 h after indicated treatments (Cys: 250 nM, Aur: 0.5 μM, GSH: 2 mM; data from a representative experiment). Scale bars, 5 μm. **c** Confocal microscopy images of mCherry-GFP-LC3-transfected MIA-PaCa2 cells 6 h after indicated treatments (Cys: 250 nM, Aur: 0.5 μM, Bafilomycin A1: 10 nM; data are from a representative experiment). Scale bars, 5 μm. **d** Mitochondrial mass was analyzed by labeling MIA-PaCa2 cells with MitoTracker Green. **e** Quantification of MitoTracker Green fluorescence after indicated combinatorial treatments for 24 h (*n* = 3–4 independent experiments). **f** Mitochondrial membrane potential (Δψ_m_) was analyzed by labeling MIA-PaCa2 cells with MitoTracker Green and MitoTracker Red. **g** Quantification of cells with defective mitochondrial membrane potential (Δψ_m_) after indicated combinatorial treatments for 24 h (*n* = 4 independent experiments). All data represent mean ± s.e.m. For (**e**, **g**), Cys: 50 nM for MIA-PaCa2 and Panc1, 100 nM for BxPC3; Aur: 0.5 μM for MIA-PaCa2 and Panc1, 2 μM for BxPC3; ####*P* or ****P < 0.0001; compared to untreated controls (*, comparison done for Cys, Aur and Cys + Aur) or to Cys + Aur (#, comparison done for only Cys + Aur + GSH); two-way ANOVA with Bonferroni’s method for multiple-comparison test

As noted above, the protein level of thioredoxin 1 was positively correlated with overall sensitivity to cyst(e)inase treatment among the three pancreatic cancer cell lines (Fig. 4a, c). Thioredoxins are antioxidant proteins that scavenge ROS by cycling between oxidized and reduced forms with the help of thioredoxin reductases. Mammalian cells have two major isoforms—a cytosolic thioredoxin 1 and a mitochondrial thioredoxin 2 that pair up with thioredoxin reductases 1 and 2 (TXNRD1 and TXNRD2), respectively.^34,35^ Auranofin inhibits both isoforms of thioredoxin reductase^36^ and is also an approved drug (Ridaura®) for the treatment of rheumatoid arthritis. Notably, auranofin treatment sensitized both MIA-PaCa2 and BxPC3 to cyst(e)inase treatment (Fig. 4f). The synergistic decrease in cell survival was paralleled with synergistic increases in mROS and cell death, which was rescued by supplementation with cell-permeable GSH (Fig. 4g, h). Growth-promoting cell cycle checkpoint proteins (phosphorylated retinoblastoma, cdk1, cdc25C) were decreased and the inhibitory cell cycle protein p27 was increased by the combination of cyst(e)inase and auranofin (Fig. 4i). Parameters of oxidative stress and DNA damage (activation of ATM and H2AX, and upregulation of c-Jun via activation of JNK) as well as markers of apoptosis (cleavage of ATM, PARP, and caspases 3 and 7) were increased by the combination treatment and rescued by GSH supplementation in all three cell lines. Figure 5a shows the results for MIA-PaCa2 cells and similar results were obtained for the other two cell lines. mROS induced by the combination was capable of reaching the nucleus to cause the observed genotoxic effects (Supplementary Fig. 5a, b).

We also investigated the effect of combination treatment on autophagy given its role in mitochondrial quality control and found an inhibition of autophagic flux as indicated by accumulation of both forms of LC3 protein as well as p62 (Fig. 5a). MIA-PaCa2 cells transfected with an mCherry-GFP-LC3 construct demonstrated that this autophagic defect is due to failure of autolysosome formation, evidenced by an increase in colocalization of red and green fluorescence (yellow puncta), which was also seen in cells treated with Bafilomycin A1, an inhibitor of autophagosome and lysosome fusion^37^ (Fig. 5b, c). This block in autophagy caused an accumulation of defective mitochondria that cannot maintain membrane potential (Fig. 5d–g), which are more prone to producing ROS.^38^ Since GSH supplementation rescued this inhibition of mitophagy (Fig. 5a, b, d–g), ROS stress appeared to be the inciting event which supports the idea that the cyst(e)inase and auranofin combination is likely causing a vicious cycle of ROS stress and accumulation of defective mitochondria. In addition to oxidative damage, the loss of mitochondrial fitness abrogates the ability of cyst(e)inase-resistant cells to survive under l-Cys/CSSC deprivation. To further explore the effects of auranofin, we assessed the extent of inhibition required in thioredoxin reductase (TxnR) activity to produce the observed synergy with cyst(e)inase. Surprisingly, only after achieving more than 50% inhibition of TxnR activity, was auranofin able to appreciably synergize with cyst(e)inase suggesting a considerable redundancy in the thioredoxin antioxidant system (Supplementary Fig. 6a, b). Additional evidence validating thioredoxin reductase as the major mechanistic target producing synergy with the cyst(e)inase and auranofin combination was provided by the fact that natural compound inhibitors of thioredoxin reductase (curcumin, myricetin, and quercetin^39,40^) also produced a synergistic inhibition of cell survival when combined with cyst(e)inase (Supplementary Fig. 6c, d).

### Cyst(e)inase plus auranofin or tigecycline synergistically suppresses growth of PDAC xenografts

**Fig. 6:**
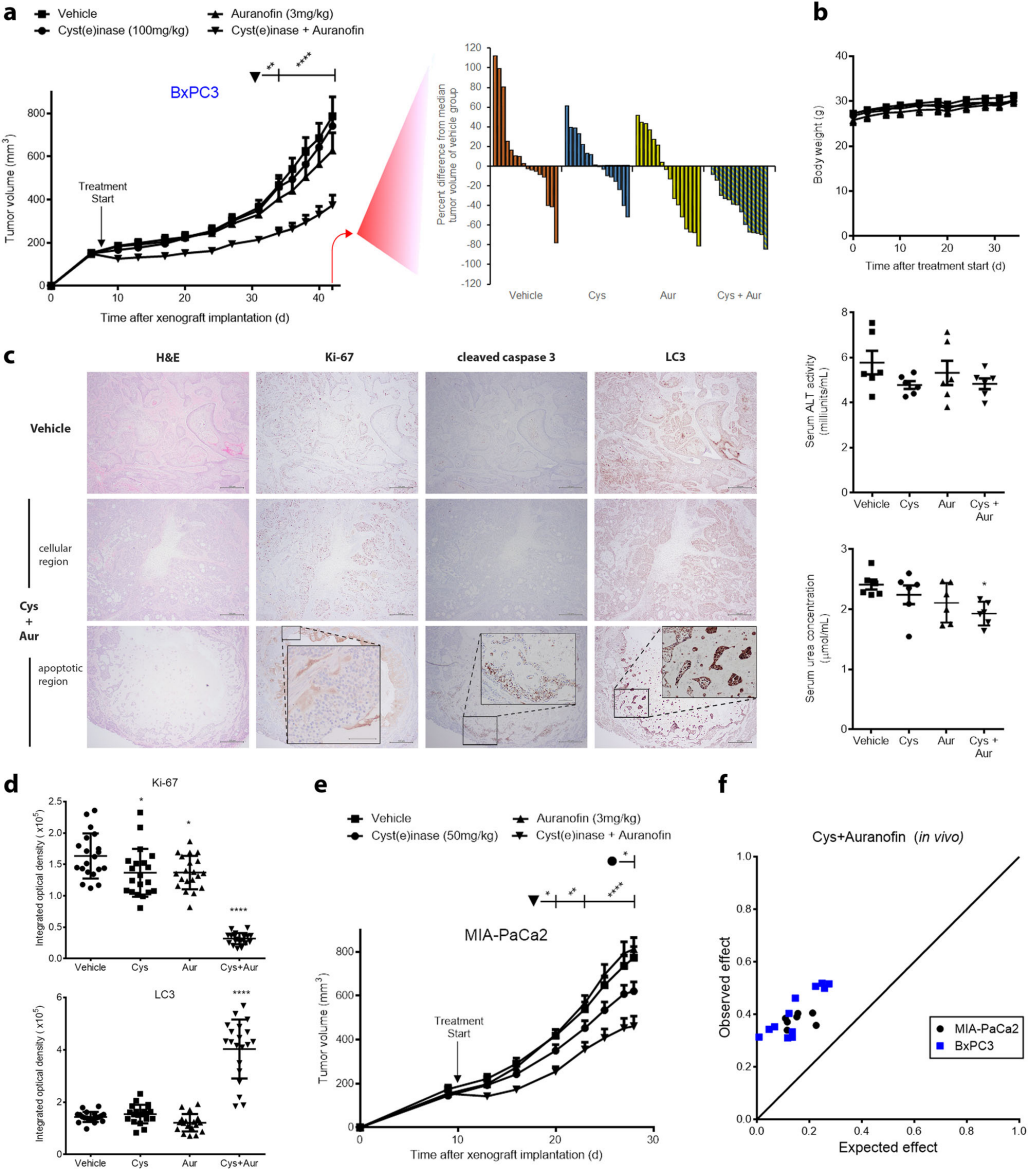
Cyst(e)inase synergistically inhibits growth of pancreatic cancer xenografts in combination with auranofin without toxicity. **a** Growth of xenografted BxPC3 pancreatic tumors in male nude mice treated with vehicle control (*n* = 8 mice), cyst(e)inase (Cys, *n* *=* 8 mice), auranofin (Aur, *n* *=* 8 mice), or cyst(e)inase and auranofin in combination (*n* *=* 7 mice), and waterfall plots indicating the percent difference from median tumor volume of vehicle-treated group at day 42. **b** Average body weight (top), liver toxicity as assessed by serum alanine aminotransferase (ALT) activity (middle), and renal function as assessed by serum urea concentration (bottom) in mice from **a**. **c**, **d** Representative immunohistochemical staining **c** of xenografts from **a**, and their quantification **d**, which was performed in ImageJ software. Scale bars, 500 μm (Inset: 100 μm). **e** Growth of xenografted MIA-PaCa2 pancreatic tumors in male nude mice treated with vehicle control (*n* = 8 mice), cyst(e)inase (*n* *=* 8 mice), auranofin (*n* *=* 9 mice), or cyst(e)inase and auranofin in combination (*n* *=* 9 mice). **f** Isobologram of the effect of the combination of cyst(e)inase (Cys) and auranofin on BxPC3 and MIA-PaCa2 xenografts. All data represent mean ± s.e.m. *P < 0.05, **P < 0.01, ****P < 0.0001; compared to vehicle controls; repeated-measures two-way ANOVA (**a**, **e**) or two-way ANOVA (**b**, **d**) with Bonferroni’s method for multiple-comparison test

To further assess the potential clinical relevance of our findings, additional preclinical studies were performed using cyst(e)inase in combination with either auranofin or tigecycline in xenograft models. Notably, co-administration of auranofin (3 mg/kg) and cyst(e)inase (100 mg/kg) markedly inhibited the growth of BxPC3 xenografts whereas either agent alone had no significant effect at the doses examined (Fig. 6a). Importantly, the combination did not produce any overt signs of systemic toxicity as body weight stayed constant and there was no increase in serum ALT (alanine aminotransferase) activity or urea concentration, markers of liver and kidney toxicity, respectively (Fig. 6b). Immunohistochemical analyses showed that the combination treatment produced focal areas of apoptotic cells (cleaved caspase 3) that exhibited reduced proliferation (Ki67) and reduced mitophagy (LC3 accumulation), which mirrored changes seen in cultured cells treated with the combination (Fig. 6c, d). The combinatorial effect observed with cyst(e)inase and auranofin in BxPC3 xenografts was also observed in MIA-PaCa2 xenografts (Fig. 6e). The inhibitory effects of the combination of auranofin and cyst(e)inase on both BxPC3 and MIA-PaCa2 xenograft tumor growth were synergistic (Fig. 6f).

To further confirm the role of mitochondrial metabolism in resistance to cyst(e)inase treatment, the effect of combining cyst(e)inase with tigecycline on growth of BxPC3 xenografts was evaluated. As shown in Supplementary Fig. 7a, a combination of cyst(e)inase plus tigecycline at doses where neither compound alone inhibited tumor growth produced a significant and synergistic inhibition of BxPC3 tumor growth. Again, there were no overt signs of toxicity with this combination as body weight (Supplementary Fig. 7b) did not differ from the control or single treatment groups.

## Discussion

In this study, we evaluated the impact of cyst(e)inase-mediated l-Cys/CSSC depletion on three pancreatic cancer cell lines and discovered differences in sensitivity with one cell line (i.e., BxPC3) completely resistant to the effects of l-Cys/CSSC depletion. Further investigation revealed that cyst(e)inase treatment caused mROS accumulation and apoptosis in only one of three pancreatic cancer cell lines examined (i.e., Panc1 cells). This was a rather surprising finding in the context of our previous study where cyst(e)inase treatment produced a marked reduction of growth in multiple other cancer models both in vitro and in vivo.^10^ Furthermore, given the fact that pancreatic cancer cells dedicate multiple aspects of their cellular machinery specifically to maintain redox homeostasis,^12,14,41^ targeting the common denominator by disrupting one of the major intracellular antioxidants in GSH was predicted to also be cytotoxic to most, if not all, pancreatic cancer cells. Upon depletion of intracellular l-Cys and GSH, the sensitive cell line (Panc1) exhibited both oxidative stress as well as compromised anaplerotic capacity. In contrast, the more resistant cell lines (MIA-PaCa2 and BxPC3) maintained mitochondrial redox balance and TCA cycle activity for the production of aspartate, both of which were integral in their ability to survive during cyst(e)inase treatment and intracellular GSH depletion. Consequently, inhibition of biosynthetic and bioenergetic functions of the mitochondria sensitized the more resistant cells to cyst(e)inase treatment. Alternately, inhibiting other antioxidant defense mechanisms, which are required to detoxify mROS produced by the electron transport chain, greatly synergized with GSH depletion resulting from treatment with cyst(e)inase. The most striking combinatorial effect on both cell survival and in vivo tumor growth was provided by auranofin, a specific inhibitor of thioredoxin reductase and an FDA-approved drug for rheumatoid arthritis. Based on the data generated in this study, a working model is presented in Supplementary Fig. 8 to explain both the differences in sensitivity to cyst(e)inase in PDAC cells, and how those differences can be overcome through combinatorial treatment approaches.

While concurrently inhibiting the GSH and thioredoxin antioxidant systems is already known to produce synergistic cell killing of cancer cells in vitro,^42,43^ using a combination of BSO and auranofin to achieve this end in vivo might not be an effective therapeutic strategy.^43^ Our results demonstrate that combining auranofin with cyst(e)inase may represent a more effective approach. An interesting mechanism uncovered in the current study through which cyst(e)inase synergizes with auranofin is by inhibition of autophagic flux. This is especially important in the setting of pancreatic cancers, which rely on autophagy in a tumor cell-autonomous and nonautonomous fashion to support growth.^14,44,45^ A major functional subset of autophagy is recycling damaged mitochondria (mitophagy), which ensures the proper functioning of oxidative metabolism as well as clearance of mROS.^13,14,38^ The pancreatic cancer cells more resistant to treatment with cyst(e)inase were able to maintain mitochondrial function and at the same time resist accumulation of mROS, both of which were abrogated by the inhibition of mitophagy precipitated by combining cyst(e)inase and auranofin. Furthermore, pancreatic cancers utilize autophagy to sustain intracellular amino acid pools via control by the MiT/TFE transcription factors;^46^ this is dysregulated by the combination treatment-mediated inhibition of autophagy and likely contributes to the cytotoxic mechanism of the drug combination. A caveat to this combination treatment is that appreciable synergy is attained only when thioredoxin reductase is near-completely inhibited. While literature precedence mostly guided our auranofin dosing decisions for xenograft studies, a comprehensive knowledge of dosing and corresponding tissue-specific inhibition levels in humans of this FDA-approved drug would maximize its potential clinical utility for cancer treatment.

For about two decades, gemcitabine has been the standard-of-care chemotherapeutic agent for pancreatic cancer even though it provides only a modest extension of survival of ~6 months.^2^ There have been a few notable combination therapies for pancreatic cancer that have produced encouraging bed side results. Nanoparticle albumin-bound (nab)-paclitaxel was found to potentiate the effects of gemcitabine by increasing the intratumoral concentration of gemcitabine in preclinical studies.^47^ Subsequently, a multicenter phase III clinical trial demonstrated the improved efficacy of this combination (8.5 months) compared to gemcitabine alone (6.7 months).^48^ The greatest increase in survival in advanced PDAC to date has been achieved with FOLFIRINOX (a combination of folinic acid, 5-fluorouracil, irinotecan and oxaliplatin) which increases survival to 11.1 months; however, this treatment regimen is only reserved for patients with good performance due to its higher incidence of toxicity.^49^ The need for more effective therapy is clear, but far too often effectiveness and systemic toxicity cannot be deconvoluted. We have previously shown that long-term cyst(e)inase treatment does not produce significant adverse effects in mice.^10^ Our current data demonstrate that coadministration of cyst(e)inase and auranofin can synergistically suppress the growth of pancreatic cancer cells with various genetic backgrounds in vitro as well as in vivo, and has a favorable safety profile to justify further clinical evaluation.

## Methods

### Reagents

CB-839, Quercetin, Myricetin (Selleckchem); Glutathione ethyl ester, Buthionine sulfoximine, Curcumin, Bafilomycin A1 (Sigma); Tigecycline, Rotenone, Oligomycin (Cayman); UK5099 (Tocris); Sulfasalazine (Fluka); Auranofin (Adipogen); l-aspartic acid 4-methyl ester hydrochloride (Alfa Aesar) were used.

### Cell lines and culture

Human pancreatic cancer cell lines MIA-PaCa2, Panc1 and BxPC3 were purchased from the American Type Culture Collection (ATCC; Manassas, VA). Cell lines were confirmed to be mycoplasma free by PCR amplification (Applied Biological Materials Inc.) and 4,6-diamidino-2-phenylindole (DAPI) staining. Low passage cells (*P* < 30) were cultured in either DMEM (MIA-PaCa2 and Panc1; Life Technologies) supplemented with 10% fetal bovine serum (FBS; Life Technologies) and 1% penicillin/streptomycin (Pen/Strep; Life Technologies) or RPMI-1640 (BxPC3; Life Technologies) supplemented with 10% FBS, 1% Pen/Strep, 1 mM sodium pyruvate (Corning) and 1 mM HEPES (Sigma). All cells were cultured at 37 °C in 95% air and 5% CO_2_. Cystine-deficient DMEM (Life Technologies, Cat. #21013024) and RPMI (Corning, Cat. #17104CI) were prepared by supplementing the base media with the aforementioned reagents and deficient nutrients except cystine.

### Western blotting

After treatment with indicated agents for the specified time, media was collected and centrifuged to pellet floating cells, and adherent cells were washed with PBS. Both cell fractions were combined and lysed in RIPA buffer with 1× protease and phosphatase inhibitor cocktails (Sigma). Protein concentration was quantified using DC Protein Assay (Bio-Rad) with bovine serum albumin (BSA) as a standard. Equal amounts of protein were separated on either fixed percentage or 4–15% gradient SDS-PAGE gels and transferred to 0.45 μm nitrocellulose membranes (Bio-Rad). After blocking in 3% BSA for 30 min, membranes were probed with specific primary antibodies (listed below) overnight at 4 °C and secondary antibody at room temperature for 2 h. Membranes were visualized with chemiluminescent detection kits (SuperSignal West Pico, Thermo Scientific for stronger protein targets; WesternBright Quantum, Advansta for weaker ones). For all western blots shown, samples were derived from the same experiment and processed in parallel.

The following primary antibodies were used (diluted 1:1000 in 3% BSA, except where indicated): p-JNK^Thr183/Tyr185^ (9251), PARP (9542), p-eIF2α^Ser51^ (3398), ATF4 (11815), p-p70S6K^Thr389^ (9234, 1:500), p-S6 ribo^Ser235/236^ (2211, 1:5000), S6 ribo (2217, 1:5000), p-Rb^Ser807/811^ (8516), Rb (9309), Cyclin D3 (2936), cdc25C (4688), p-AMPK^Thr172^ (2531, 1:500), AMPK (2532), xCT (12691), Thioredoxin 1 (2429), Thioredoxin reductase 1 (15140), Thioredoxin 2 (14907), Thioredoxin reductase 2 (12029), c-jun (9165), Cleaved caspase 3 (9664, 1:500), Cleaved caspase 7 (9491, 1:500), LC3B (3868), p62 (8025), Hexokinase II (2867), pyruvate dehydrogenase (2784) from Cell Signaling; Sestrin2 (sc-393195), Cyclin B1 (sc-752), Nrf2 (sc-13032, 1:500), Cdk1 (sc-54), p27 (sc-776), succinate dehydrogenase-subunit A (sc-390381) from Santa Cruz Biotechnology; p-ATM^Ser1981^ (ab81292), GCL-C (ab53179) from Abcam; p-H2AX^Ser139^ (05-636, Millipore) and Actin (A5316, Sigma, 1:20,000). The following secondary antibodies were used (diluted 1:5000 in 5% milk): HRP-linked Anti-mouse IgG (NA931V) and HRP-linked Anti-rabbit IgG (NA934V) from GE Healthcare.

### Mouse studies

For xenograft studies, one (MIA-PaCa2), two (BxPC3) or five (Panc1) million cells in a 200 µL solution of Matrigel (Corning) and serum free media (1:1) were injected subcutaneously into the hind flanks of 5–6-week-old athymic male nude mice (outbred homozygous *Foxn1*^*nu*^*/ Foxn1*^*nu*^; J:NU 007850, Jackson Laboratory). After tumor volumes reached a size of ~150 mm^3^ (as calculated using the formula 0.5 × *l* × *w*^2^, where *l* and *w* represent long and short diameters respectively, and are measured using a digital caliper), mice were divided into groups such that average tumor volumes in all the groups were approximately equal and treatment with indicated agents was begun as following: for single-agent cyst(e)inase studies, mice were given intraperitoneal (i.p.) injections twice a week with either 100 mg/kg cyst(e)inase or PBS. For combination studies, cyst(e)inase (50 or 100 mg/kg) was administered i.p. twice a week, auranofin (3 mg/kg) was administered i.p. three times a week, and tigecycline (30 mg/kg) was administered i.p. 3 times a week. Control mice were injected with PBS on cyst(e)inase treatment days and a 1:1 solution of 10% polyethylene glycol and 10% Tween80 in water (the solvent that was used to dilute 25 mg/mL DMSO-dissolved auranofin to make the 0.5 mg/mL auranofin solution used for treatment). Mice were given a semi-purified diet (AIN76A, 10 kcal%, Research Diets) and water ad libitum. Body weight and food consumption of mice were measured triweekly and weekly respectively. Experiments were terminated when tumor sizes in the control group reached their maximum limit as specified by an approved protocol from the University of Texas at Austin Institutional Animal Care and Use Committee (IACUC). At study termination, blood was collected via cardiac puncture following euthanasia and assessed for liver and kidney function by measuring serum levels of alanine aminotransferase and urea respectively using commercially available kits (Sigma).

### Intracellular ROS measurement

Cells were seeded at a density of 1 × 10^5^ (3.5-cm dish) or 5 × 10^5^ (6-cm dish) and allowed to attach for ~24 h. Following treatment with different agents for indicated times, 2′,7′-dichlorofluorescein diacetate (DCFDA, Sigma) for measuring total cellular ROS and MitoSOX Red mitochondrial superoxide indicator (Invitrogen) were added directly to the media at 20 and 2 µM final concentration respectively, and incubated at 37 °C for 30 min. Cells were trypsizined, resuspended in PBS and data were acquired by flow cytometry (Guava easyCyte 8HT, EMD Millipore) and analyzed with FlowJo software.

### Cell viability, cell growth and cell death assay

Cells were plated in 96-well plate at 5000 cells per well (100 µL). After ~24 h of attachment, cells were treated with 100 µL of different agents at 2× desired concentration for the indicated time points. At the end of treatment, media was aspirated and remaining viable cells were fixed with 10% formalin and stained with 0.05% crystal violet. After washing with water once, dye was extracted with 10% acetic acid and absorbance at 595 nm was measured. Relative cell viability was computed by comparing absorbance to untreated or vehicle-treated cells. Cell growth was assessed by crystal violet staining as well. Briefly, 10–20 × 10^3^ cells were plated in 3.5-cm dish and allowed to attach for 1–2 days. At day = 0, one plate was stained with crystal violet as a surrogate measurement of cell number at the time of treatment. At every indicated subsequent time point, the relative cell number was calculated by measuring the fold change in crystal violet intensity relative to that at day = 0. Cell death was assessed by trypan blue exclusion test. Briefly, 2.5–5 × 10^5^ cells were plated at day = −1, treated with different agents at desired concentration at day = 0 and trypsinized after indicated time of treatment. Dead/floating cells were also collected. A 1:1 mixture of trypan blue dye (Bio-Rad) and the cell suspension of interest was counted using a hemocytometer or TC20 Automated Cell Counter (Bio-Rad) for live and dead (blue) cell count.

### Measurement of mitochondrial contents

1 × 10^5^ cells were plated in 3.5-cm dishes and allowed to attach for ~24 h. Following treatment with different agents for 24 h, MitoTracker Green FM (for total mitochondrial mass; Invitrogen) and MitoTracker Red CMXRos (for mitochondrial membrane potential; Invitrogen) were directly added to the media at 50 nM and 200 µM final concentration respectively. After incubation for 30 min at 37 °C, cells were trypsinized and resuspended in PBS. Data were acquired by flow cytometry (Guava easyCyte 8HT, EMD Millipore) and analyzed with FlowJo software as previously described.^38^

### Intracellular glutathione measurement

Following treatment of 1 × 10^5^ cells for 24 h, media was aspirated, cells were washed with PBS, and pelleted and mixed with 150 µL of 5% sulfosalicylic acid (Sigma). Cells were lysed via two cycles of freeze-thaw, cell debris was pelleted by centrifugation and the glutathione content in the resulting supernatant was measured using a glutathione detection kit (Sigma). Separate dishes that were treated in parallel were used to quantify protein content, which was then used for glutathione normalization. Alternately, intracellular glutathione content was also measured in a 96-well format using the GSH/GSSG-Glo Assay (Promega) following a 24 h treatment with normalization performed to viable cell count.

### Cell cycle analysis

After treatment of 5–8 × 10^5^ cells with different concentrations of cyst(e)inase for indicated time points, adherent and floating cells were harvested, washed with PBS and fixed in ice-cold 70% ethanol at 20 °C for 2–4 days. After fixation, cells were washed, resuspended in Hank’s Balanced salt solution (HBSS) containing RNase A (100 µg/mL; Thermo Scientific) and propidium iodide (PI; 40 µg/mL, Invitrogen) and incubated for 30 min at 37 °C. Data of cell-cycle phase distribution were acquired by flow cytometry and analyzed by FlowJo software.

### Isobologram and evaluation of synergy

To evaluate synergism between two compounds we used the Bliss Independence Model as previously described.^50,51^ Briefly, the expected effect (*E*_exp_) in a combination of two single agents is calculated from the effects of each separate agent as follows:

1 $$E_{{\mathrm{exp}}} = E_{{\mathrm{agent}}1} + E_{{\mathrm{agent}}2}-\left( {E_{{\mathrm{agent}}1} \times E_{{\mathrm{agent}}2}} \right).$$

The Bliss index of a combination is the ratio of the observed effect to the expected effect with values = 1, <1, or >1 indicating additivity, antagonism and synergy respectively. Experimental data points that fall in the upper left region of an isobologram showing correlation of observed vs. expected effects indicate increasing level of synergy.

### Polar metabolite extraction and GC-MS analysis

5 × 10^5^ cells were plated in 6-cm dishes and allowed to attach for ~24 h. After treatment with cyst(e)inase in regular media for the indicated time points, media was aspirated and cells were washed twice with 0.9% saline solution. Polar metabolites were extracted and analyzed by GC-MS as previously described.^24^ Cells were scraped in 500 µL of a mixture of Optima HPLC grade methanol (Fisher Scientific) and water (1:1) solution, transferred to a tube and snap frozen in liquid N_2_ until further use. Cells were lysed via three cycles of freeze-thaw and cell debris was pelleted by centrifugation (14.8 × 10^3^ rpm, 10 min, 4 °C). The resulting supernatant was transferred to a glass vial, mixed with an equal volume of ice-cold Optima grade chloroform (Fisher Scientific), vortexed and centrifuged in an Allegra X-15r Centrifuge (Beckman Coulter) for 20 min at 4 °C for polar-nonpolar phase separation. The top polar phase was transferred to a new tube and dried at 4 °C using refrigerated CentriVap Concentrator attached to −84 °C CentriVap Cold Trap (Labconco) and MD 4C NT vacuum pump (Vacuubrand). Metabolites were derivatized by incubation at 37 °C for 1 h in 20 µL of methoxyamine hydrochloride (Sigma, 20 mg/mL dissolved in pyridine) then in 30 µL of *N*-methyl-*N*-(trimethylsilyl)trifluoroacetamide with 1% trimethylchlorosilane (Sigma) for another 1 h at 37 °C. A small volume (0.2 µL) of derivatized samples were injected via split-less injection into a Thermo Scientific Trace 1310 gas chromatograph loaded with a Thermo TR-5 fused silica capillary column (length = 30 m, I.D. = 0.25 mm, film = 0.25 μm), which was connected to a Thermo ISQ single quadrupole mass spectrometer (MS). Ultra-high purity helium (Praxair) at a flow rate of 1.10 mL/min was used as a carrier gas. Optima HPLC grade methanol was used to wash the injection syringe between each sample. Each sample underwent a ramp in the Trace 1310 starting at 70 °C at a rate of 3.7 °C/min until reaching 225 °C at 45 min. The MS transfer line was kept at 300 °C and the ion source was maintained at 250 °C. Electron ionization at 70 eV and a scan time of 0.25 s over the range of 60.0–650.0 amu was sufficient for analysis. Total ion current peaks of different metabolites were normalized to those of internal standard norvaline and isoleucine to control for differences in derivatization/injection and cell mass, respectively.

### [U-^13^C]-glucose labeling

Glucose that is ^13^C-labeled in all six carbons ([U-^13^C]-glucose) was purchased from Cambridge Isotope Laboratories. For all ^13^C labeling experiments, glucose and glutamine-free DMEM (Life Technologies, Cat #A1443001) was supplemented with 25 mM [U-^13^C]-glucose and 4 mM unlabeled glutamine, 10% dialyzed FBS and 1% Pen/Strep. Cells were plated at a density of 5 × 10^5^ cells (6-cm dish) in regular media. After allowing ~24 h for attachment, cells were treated with ^13^C-labeled media containing either vehicle (10% glycerol in PBS) or cyst(e)inase. After 6 h of treatment, polar metabolites were extracted and analyzed using gas chromatography-mass spectrometry (GC-MS) as mentioned above. The abundance of the following ions was used for analysis of ^13^C label incorporation: m/z 245–249 for fumarate, m/z 335–339 for malate and m/z 334–338 for aspartate. Mass isotopologue distribution for each metabolite was corrected for natural abundance of ^13^C using the software Metran.^52^

### Fluorescence microscopy for mitochondrial ROS

Cells were plated at a density of 3–5 × 10^3^ in eight-well cell culture chamber slides and allowed to attach for ~24 h. After treatment for indicated time, MitoTracker Green FM was directly added to the media at 50 nM final concentration to stain mitochondria 30 min prior to the following steps. Media was dumped into a waste jar as opposed to aspiration, which caused cells to detach. Cells were washed with PBS, the waste was dumped and any extra liquid on the slide was absorbed carefully using Kimwipes. A small volume of Vectashield mounting media containing DAPI (Vector Laboratories Inc.) was placed on each chamber and a glass cover slip was placed on top such that the media covered the entire chamber. Microscopy was performed with Olympus BX60 fluorescence microscope, and image acquisition was performed with the software DP Controller (Olympus).

### mCherry-GFP-LC3

The plasmid (Addgene, plasmid #22418) was purified from *E. coli* and transfected into MIA-PaCa2 cells using Lipofectamine 3000 (Invitrogen). After 2–4 days, cells were incubated in media containing 1 µg/mL puromycin. Surviving cells expressing both green and red fluorescence were selected and expanded. Puromycin was used for passaging of transfected cells but not during experiments. After treatment for indicated time, media was dumped, cells were washed with PBS, fixed with 4% paraformaldehyde solution and washed again with PBS. A small volume of Vectashield mounting media containing DAPI was placed on each chamber and a glass cover slip was placed on top. Microscopy was performed with Leica TCS SP5 II confocal microscope, and image acquisition was performed with the software Leica Application Suite (Leica Microsystems).

### Measurement of thioredoxin reductase activity

Total thioredoxin reductase activity was measured in 15 μg of protein sample using a Thioredoxin Reductase Assay kit (Cayman) according to the manufacturer’s instructions.

### Measurement of NAD+/NADH

Cells were plated in 96-well plate at 5000 cells per well (100 µL). After ~24 h of attachment, cells were treated with 100 µL of cyst(e)inase at 2× desired concentration for indicated time. At the end of treatment, NAD+/NADH was measured according to the NAD/NADH Glo Assay instructions provided by the manufacturer (Promega). Briefly, media was aspirated and replaced with 50 µL of PBS. Cells were lysed by adding 50 µL of 0.2 N NaOH with 1% dodecyltrimethylammonium bromide (DTAB) and shaking for 5 min. Fifty microliters of this cell lysate was moved to a different well and treated with 25 μL of 0.4 N HCl (acidic conditions selectively degrade NADH). NAD+ is selectively degraded in the basic conditions of the original wells. Plate was incubated for 30 min at 60 °C, allowed to equilibrate to room temperature and then neutralized with 25 μL of 0.5 M Tris base (acid-treated wells) or 50 μL of 1:1 HCl/Tris solution (0.5 M Tris + 0.4 N HCl, base-treated wells). 20 μL of this final solution was moved to a white-walled plate, mixed with 20 μL NAD/NADH-Glo™ Detection Reagent, shaken for 2 h at room temperature and the luminescence was measured.

### Statistical analyses

No statistical methods were used to predetermine sample size; selection of group sizes for animal experiments were driven by prior experience and literature precedence. The experiments were not randomized, and the investigators were not blinded during experiments or data analysis. Data are expressed as mean ± s.e.m. unless otherwise indicated and analyzed using GraphPad Prism 6. In xenograft experiments, outlier tumor values were identified and removed from all groups using the robust regression and outlier removal (ROUT) method with a 2% false discovery rate (FDR). Usage of the following statistical tests as applicable are described in figure legends: two-sided Student’s *t* test; one-way, two-way and two-way repeated-measures ANOVA followed by Bonferroni’s method for multiple-comparison test. Figure legends also describe the number of times experiments were repeated with similar results and the number of experiments that data are pooled from.

## Supplementary information

Supplementary Figures

## Data Availability

The authors declare that the data supporting the findings of this study are available within the paper and its supplementary information files. Raw data are available from the author upon reasonable request.

## Supplementary information

**Supplementary information** accompanies the paper on the *npj Precision Oncology* website (10.1038/s41698-019-0088-z).
