# Supplementary material for: Enzyme-mediated depletion of l-cyst(e)ine synergizes with thioredoxin reductase inhibition for suppression of pancreatic tumor growth
Source: NPJ Precis Oncol. 2019 Jun 3;3:16. doi: 10.1038/s41698-019-0088-z (PMC6546752; doi:10.1038/s41698-019-0088-z)
Supplement: Supplementary file 1 — Supplementary Figures [file 41698_2019_88_MOESM1_ESM.pdf]

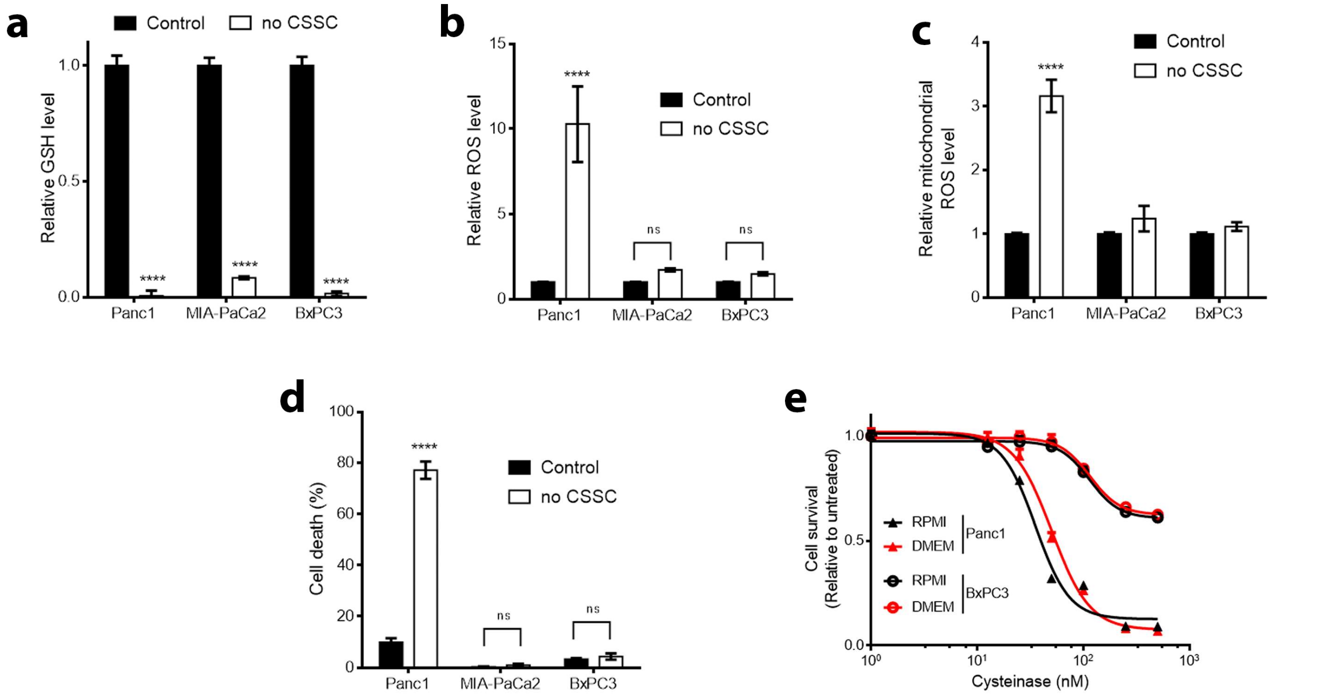

Supplementary Fig. 1 Cystine deficient media recapitulates effects of cyst(e)inase treatment in PDAC cells. **a and b** Intracellular GSH levels (**a**; *n* = 3 cultures) and cellular ROS levels (**b**; *n* = 4 independent experiments) 24 h after treatment with indicated media conditions. **c** Mitochondrial ROS levels 24 h after treatment with indicated media conditions (*n* = 3 independent experiments). **d** Cell death 48 h after treatment with indicated media conditions (*n* = 2 independent experiments). **e** Relative cell survival of Panc1 and BxPC3 cells 48 h after cyst(e)inase treatment in indicated cell culture media (*n* = 3 cultures for each dose). All data represent mean ± s.e.m. ****$P < 0.0001$; compared to untreated controls; two-way ANOVA with Bonferroni's method for multiple-comparison test. Experiments in **a** and **d** were performed once and twice respectively. All other experiments were repeated 3 times or more.

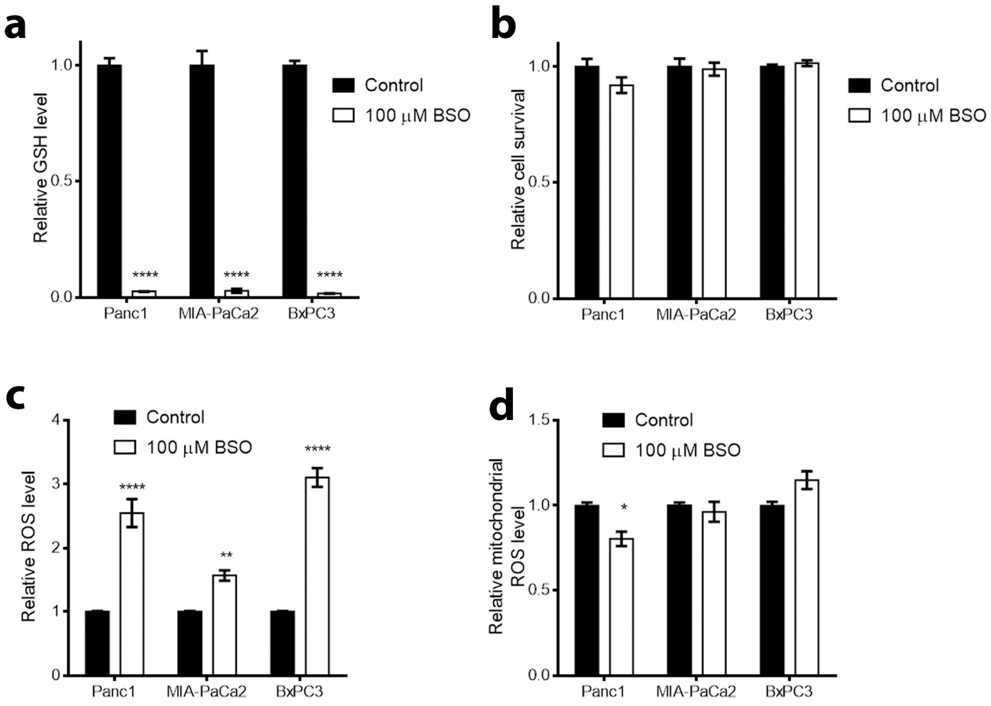

Supplementary Fig. 2 Buthionine sulfoximine (BSO), a GSH synthesis inhibitor, does not recapitulate all the effects of cyst(e)inase in pancreatic cancer cells. **a** Intracellular GSH levels 24 h after treatment (n = 3 technical replicates). **b** Relative cell survival 48 h after treatment (n = 3 cultures). **c** Cellular ROS levels 24 h after treatment (n = 2-3 independent experiments). **d** Mitochondrial ROS levels 24 h after treatment (n = 3-4 independent experiments). All data represent mean ± s.e.m. *$P < 0.05$, **$P < 0.01$, ****$P < 0.0001$; compared to untreated controls; two-way ANOVA with Bonferroni's method for multiple-comparison test. Experiment in **a** was performed once. All other experiments were repeated at least 2 times or more.

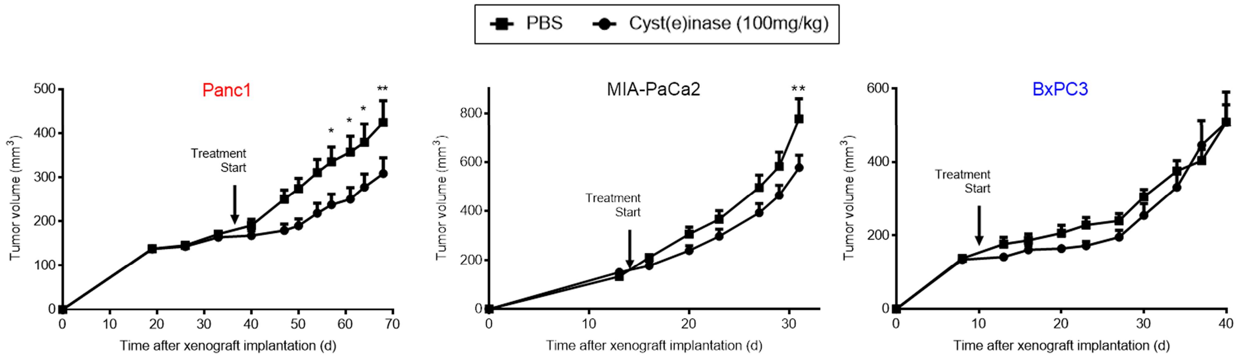

Supplementary Fig. 3 Effect of cyst(e)inase treatment on growth of pancreatic cancer xenografts Pancreatic tumors in male nude mice were treated with cyst(e)inase (*n* = 8, 7 and 8 mice for Panc1, MIA-PaCa2 and BxPC3 respectively) or PBS control (*n* = 7, 7 and 9 mice for Panc1, MIA-PaCa2 and BxPC3 respectively). All data represent mean ± s.e.m. $^{*}P < 0.05$, $^{**}P < 0.01$; compared to PBS controls; repeated-measures two-way ANOVA with Bonferroni's method for multiple-comparison test.

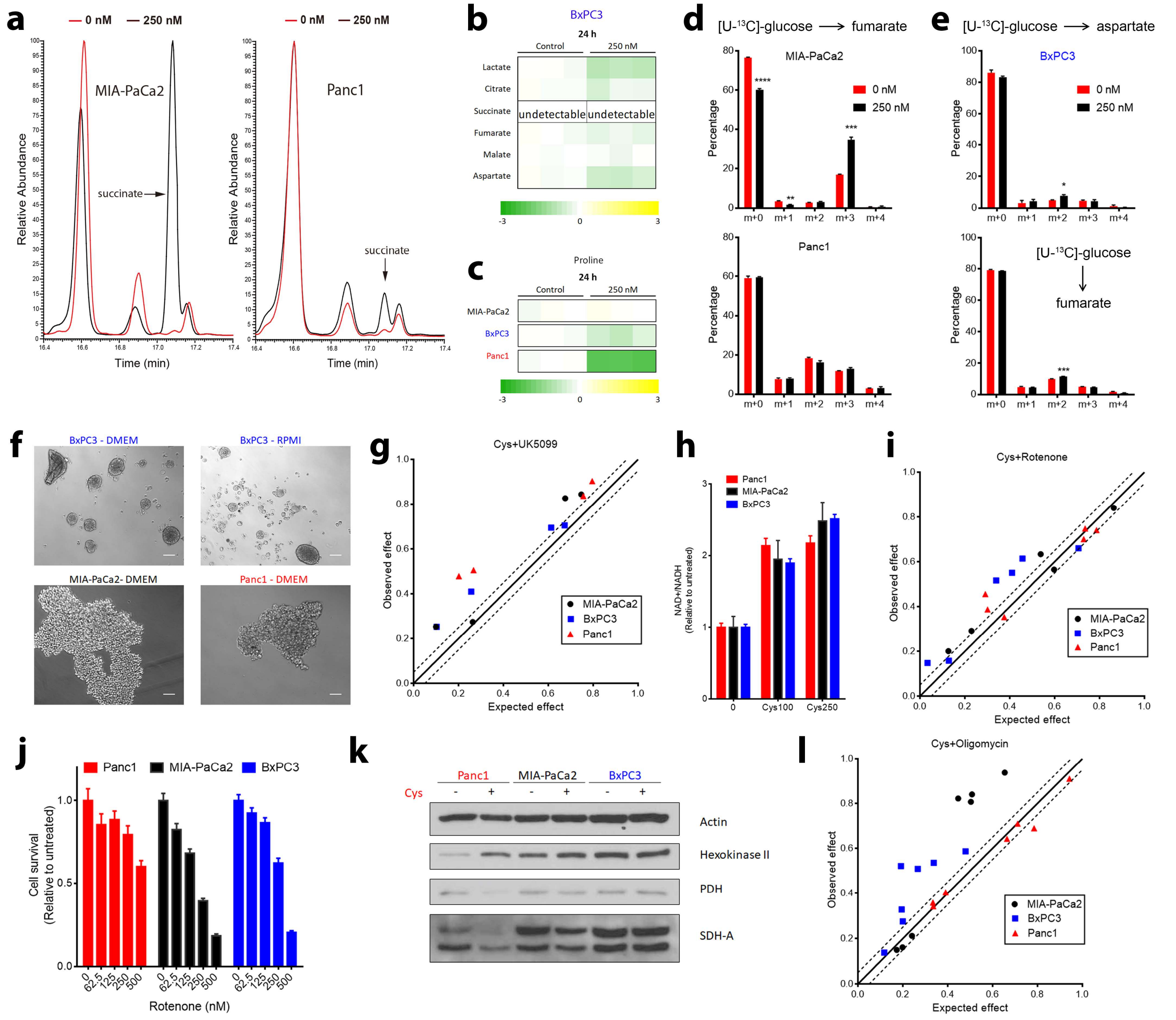

Supplementary Fig. 4 Cyst(e)inase-resistant cells are capable of maintaining biosynthetic and bioenergetic functions of the mitochondria **a** Intracellular succinate abundance in MIA-PaCa2 and Panc1 cells 3 h after cyst(e)inase treatment. **b and c** TCA cycle intermediates and related metabolite levels in BxPC3 cells (**b**; *n* = 3 cultures) and proline levels in all 3 cell lines (**c**; *n* = 3 cultures) 24 h after cyst(e)inase treatment. Color bar shows Log_2 scale. **d** Mass isotopologue analysis of fumarate in MIA-PaCa2 (top) and Panc1 cells (bottom) cultured in [U-^{13}C]-glucose and treated with cyst(e)inase for 6 h (*n* = 3 cultures). **e** Mass isotopologue analysis of aspartate (top) and fumarate (bottom) in BxPC3 cells cultured in [U-^{13}C]-glucose and treated with cyst(e)inase for 6 h (*n* = 3 cultures). **f** Spheroid formation capacity assessed 3 days after plating. BxPC3 cells were cultured in both RPMI and DMEM. Scale bars, 100 μm. **g** Isobologram of the effect of the combination of cyst(e)inase and UK5099 (data from 2 independent experiments). **h** Intracellular ratio of NAD+/NADH 24 h after cyst(e)inase treatment (*n* = 3 cultures). **i** Isobologram of the effect of the combination of cyst(e)inase and rotenone (data from 3 independent experiments). **j** Relative cell survival 48 h after rotenone treatment (*n* = 3 cultures for each dose). **k** Comparison of metabolic enzymes. "+" represents 250 nM cyst(e)inase treatment for 24 h. **l** Isobologram of the effect of the combination of cyst(e)inase and oligomycin (data from 3 independent experiments). All data represent mean ± s.e.m. **P* < 0.05, ***P* < 0.01, ****P* < 0.001, *****P* < 0.0001;

compared to untreated controls; two-sided Student's *t*-test. Experiments in **h** and **k** were performed twice. All other experiments were repeated (in exact or similar form) 3 times or more

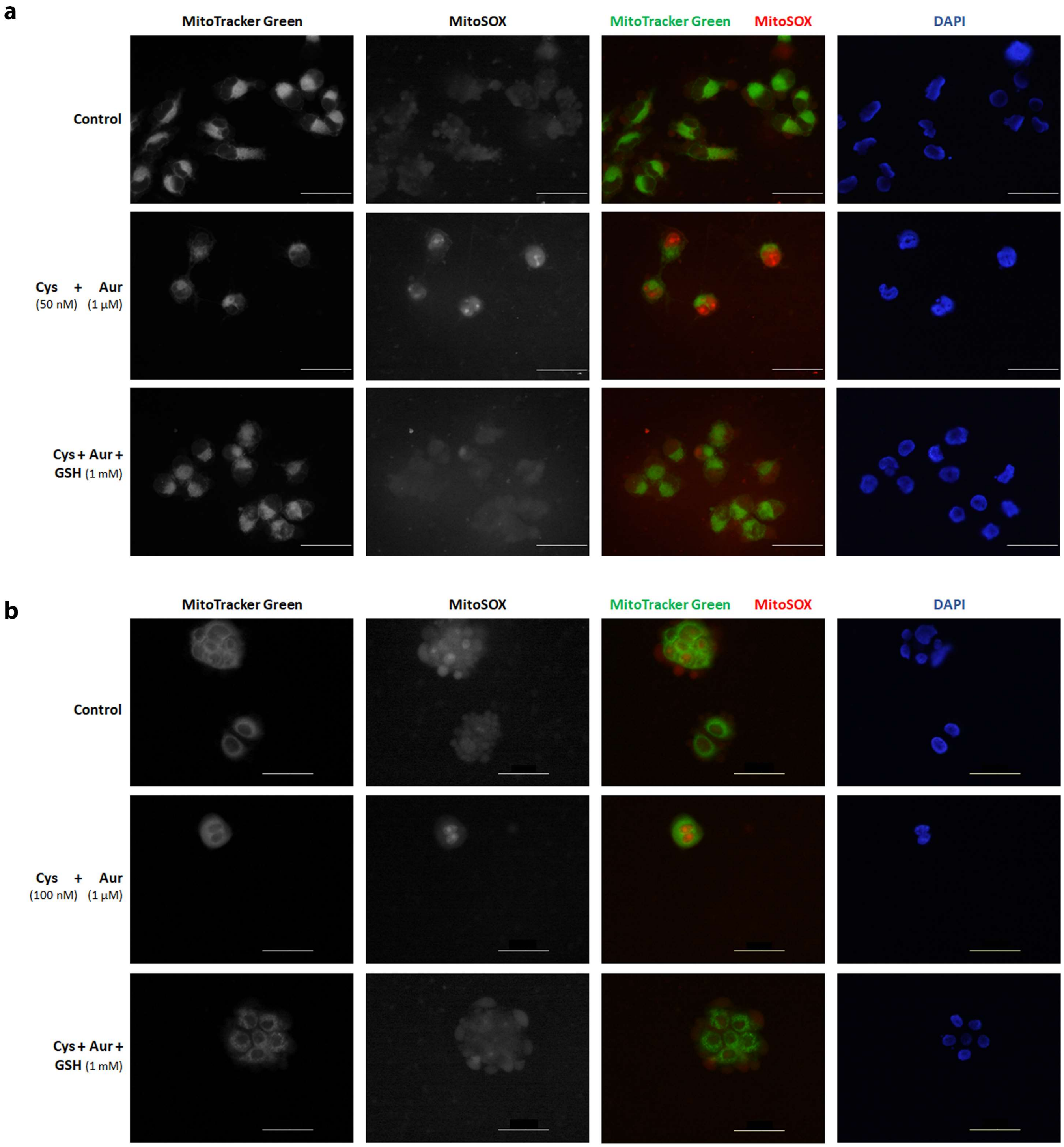

Supplementary Fig. 5 Mitochondrial ROS induced by combination treatment of cyst(e)inase and auranofin reaches the nucleus. **a and b** Representative images show mitochondrial ROS production in MIA-PaCa2 (**a**, 12 h treatment) and BxPC3 (**b**, 24 h treatment) cells labeled with MitoTracker Green and MitoSOX after indicated combinatorial treatments. Scale bars, 100 µm.

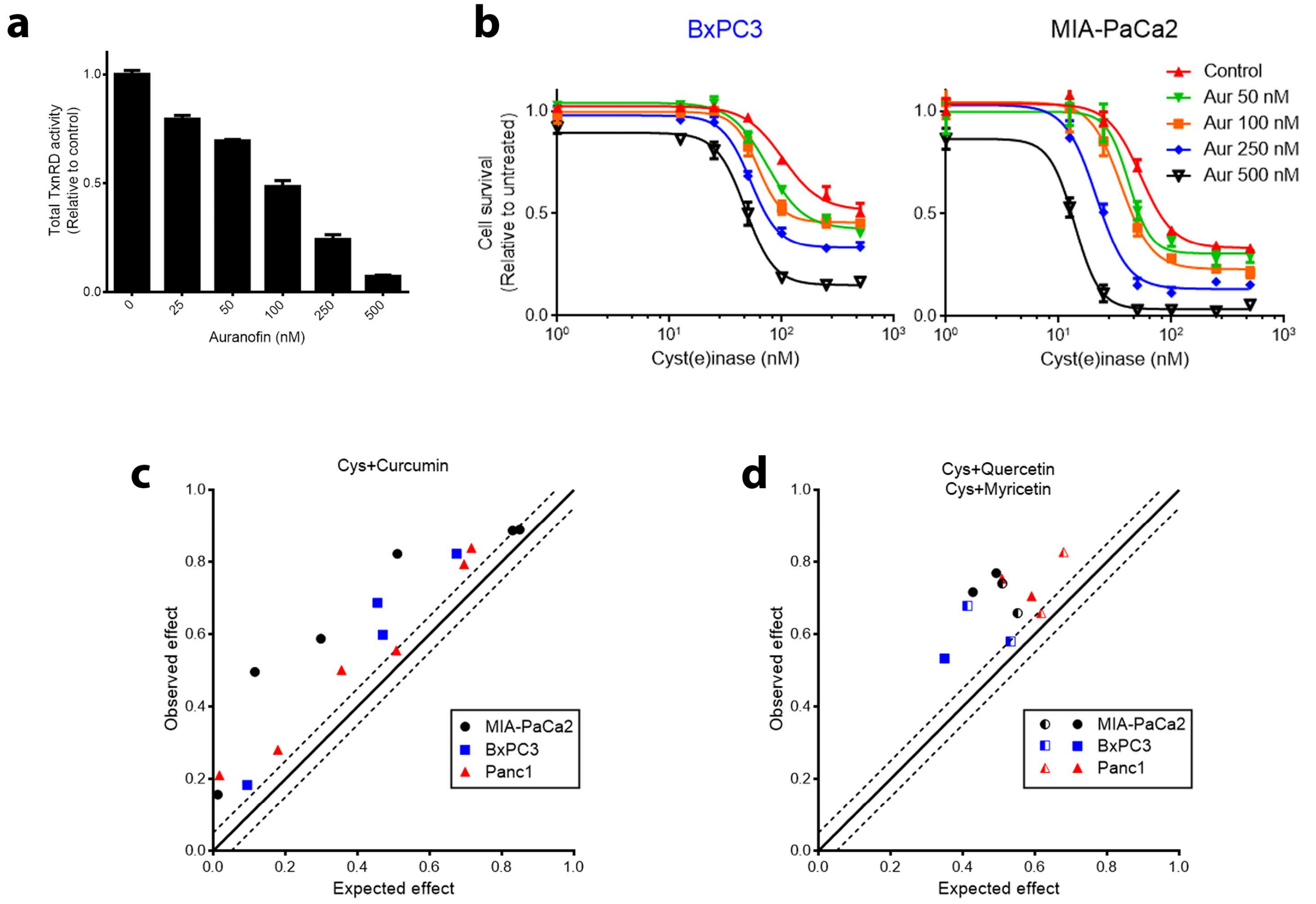

Supplementary Fig. 6 Effect of cyst(e)inase treatment in combination with inhibitors of thioredoxin reductase. **a** Total thioredoxin reductase (TxnRD) activity in MIA-PaCa2 cells treated with auranofin for 24 h (*n* = 3 technical replicates). **b** Relative cell survival 48 h after cyst(e)inase treatment in BxPC3 (left) and MIA-PaCa2 (right) cells treated concurrently with increasing concentrations of auranofin (*n* = 3 cultures for each dose). **c and d** Isobolograms of the effect of the combination of cyst(e)inase (Cys) and curcumin (**c**), Cys and quercetin (**d**, half-filled symbols), and Cys and myricetin (**d**, solid symbols) (data from 2-3 independent experiments). All data represent mean ± s.e.m. Experiment in **d** was performed twice. All other experiments were repeated 3 times.

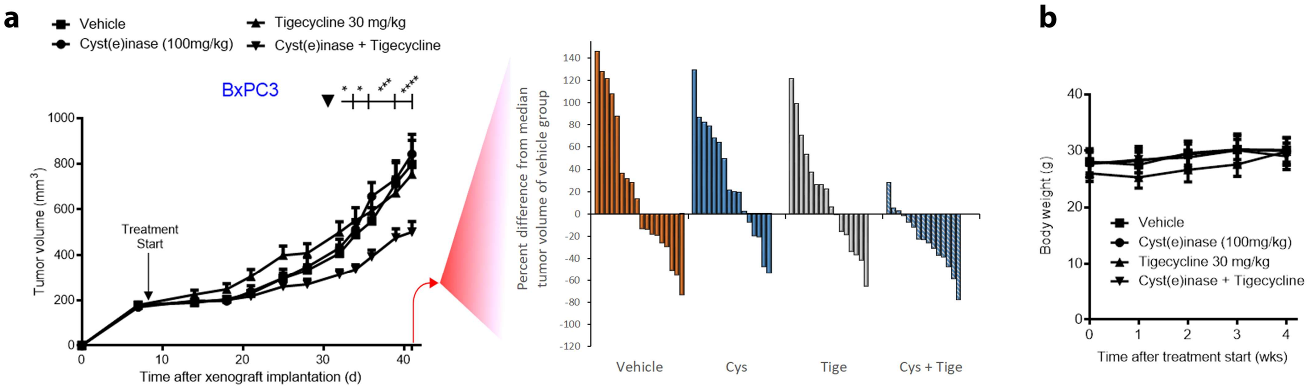

Supplementary Fig. 7 Tigecycline and cyst(e)inase synergistically inhibit growth of pancreatic cancer xenografts without toxicity. **a** Growth of xenografted BxPC3 pancreatic tumors in male nude mice treated with vehicle control (n = 9 mice), cyst(e)inase (Cys, n = 8 mice), tigecycline (Tige, n = 8 mice), or cyst(e)inase and tigecycline in combination (n = 8 mice), and waterfall plots indicating the percent difference from median tumor volume of vehicle treated group at day 41. **b** Average body weight in mice from **a**. All data represent mean ± s.e.m. *P < 0.05, ***P < 0.001, ****P < 0.0001; compared to vehicle controls; repeated-measures two-way ANOVA (**a**) or two-way ANOVA (**b**) with Bonferroni's method for multiple-comparison test.

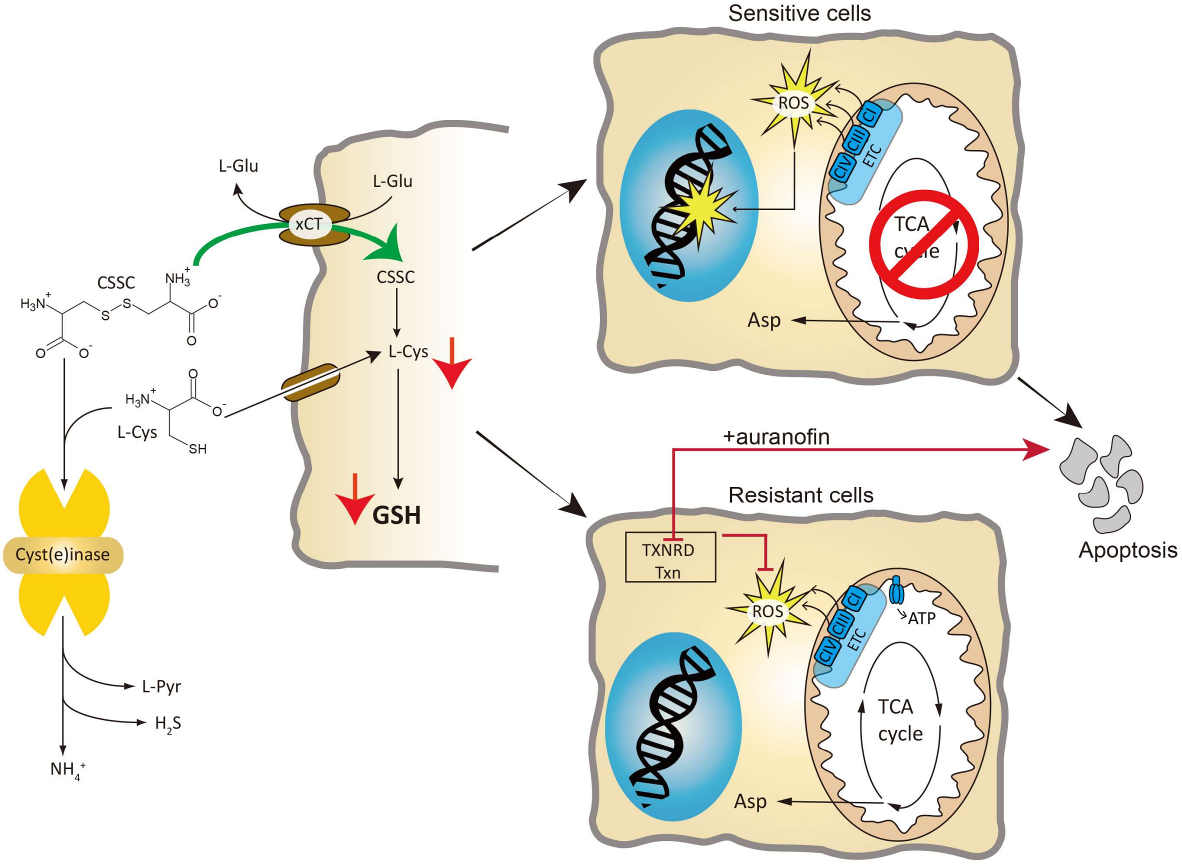

Supplementary Fig. 8 Schematic showing basis for sensitivity to cyst(e)inase in pancreatic cancer cells. Cyst(e)inase-mediated depletion of intracellular L-Cys and GSH leads to apoptosis in sensitive cells through mitochondrial dysfunction, which is associated with ROS accumulation and reduced aspartate synthesis for anabolic metabolism. Resistant cells, on the other hand, survive this deprivation by maintaining oxidative metabolism to fuel their biosynthetic and bioenergetic demands, which necessitates detoxification of ROS produced by the electron transport chain (ETC) – a liability that can be clinically targeted by auranofin, a thioredoxin reductase inhibitor.

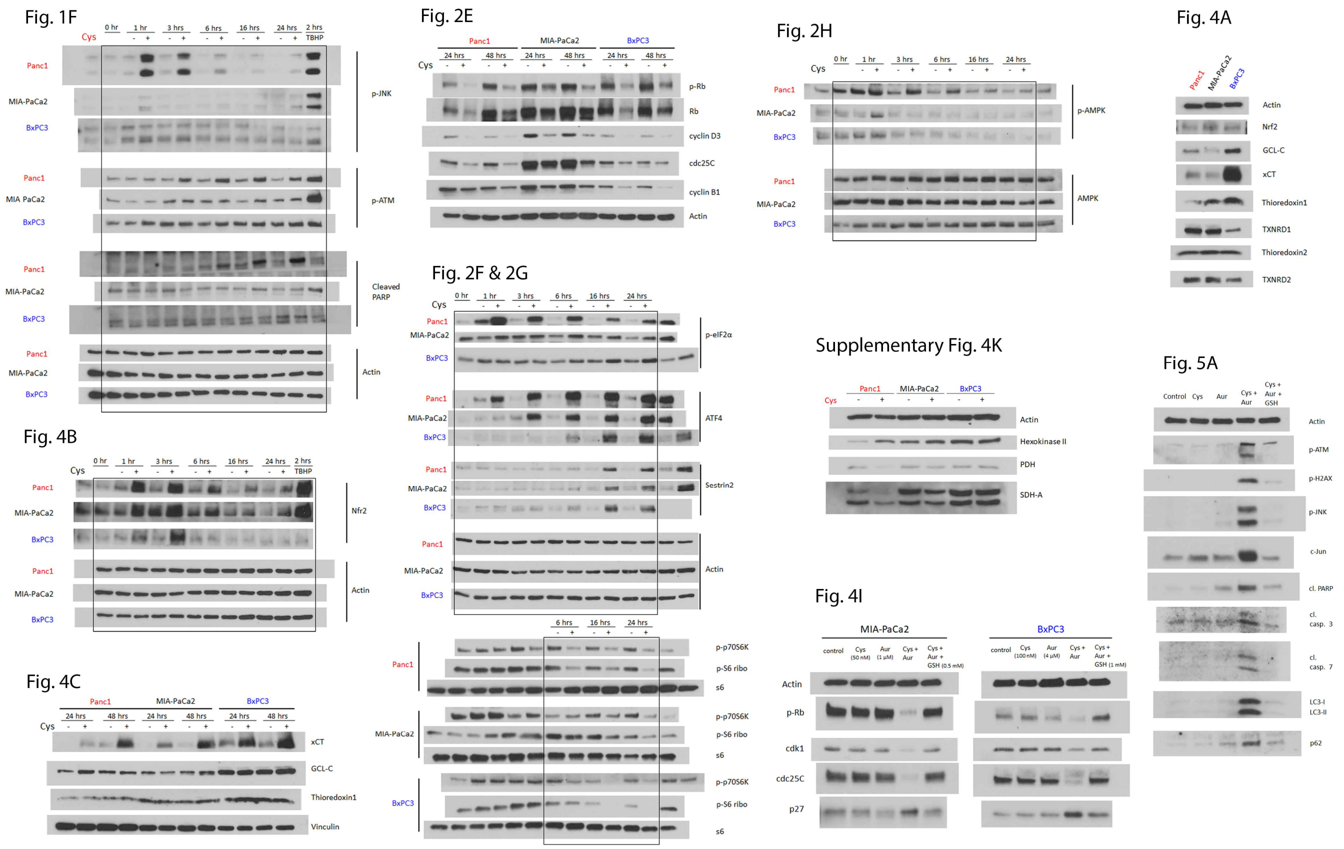

Supplementary Fig. 9 Original Western blots presented in the manuscript and supplementary information.
